# Supplementary material for: Prediction and characterisation of the human B cell response to a heterologous two-dose Ebola vaccine
Source: Nat Commun. 2025 Jul 9;16:6331. doi: 10.1038/s41467-025-61571-x (PMC12241346; doi:10.1038/s41467-025-61571-x)
Supplement: Supplementary file 1 — Supplementary Information [file 41467_2025_61571_MOESM1_ESM.pdf]

Supplementary Information

Prediction and characterisation of the human B cell response to a heterologous two-dose Ebola vaccine

Daniel O'Connor [1,2*#], Elizabeth A Clutterbuck [1,2#], Malick M Gibani[3], Sagida Bibi [1,2], Katherine A. Sanders [1,2], Rebecca Makinson [2,4], Dominic F Kelly[1,2], Andrew J Pollard[1,2]

[1] Oxford Vaccine Group, Department of Paediatrics, University of Oxford, UK

[2] NIHR Oxford Biomedical Research Centre, Oxford, UK

[3] Department of Infectious Disease, Imperial College London, St Mary's Campus, London, United Kingdom.

[4] The Jenner Institute, Nuffield Department of Medicine, University of Oxford, UK.

[#] Authors contributed equally to this work.

*Corresponding Author: Daniel O'Connor, Oxford Vaccine Group, Department of Paediatrics, Centre for Clinical Vaccinology & Tropical Medicine (CCVTM), The Churchill Hospital, Old Road, OX3 7LE, United Kingdom. Email: daniel.oconnor@paediatrics.ox.ac.uk

Supplementary Figures

	**Vaccine**		**Ad26.ZEBOV (dose 1)**						**MVA-BN-Filo (dose 2)**								PRISM EBL2001 follow up	
	Day post dose		d0	d8	d10	d12	d14		d0	d3	d5	d7	d9	d21	d180	d365	V1	V2
**EBL2001 (UK) Cohort 1 (n=30)**	Trial design/ Sample timepoints	G1						G1	d29	d32	d34	d36	d38					
		G2	d1	d9	d11	d13	d15	G2	d57	d60	d62	d64	d66					
		G3						G3	d85	d88	d90	d92	d94					
	Exploratory immunology	➢ Ex vivo B cell ELISpot (G1, G2, G3): All time points tested<br>➢ Plasma cell phenotype (G1+G3): All time points tested<br>➢ cTfh (G2 only): All time points tested																
**EBL2001 (UK) Cohort 2 (n=50)**	Trial design/ Sample timepoints	G1						G1	d29			d36		d50	d209			
		G2	d1		d11			G2	d57			d64		d78	d237			
		G3						G3	d85			d92		d106	d265			
	Exploratory immunology	➢ BMEM ELISpot (G1, G2, G3): Time points tested: d1, d29/d57/d85, d50/d78/d106, d209/d237/d265<br>➢ B cell Phenotyping (G1, G2, G3): Time points tested: d1, d29/d57/d85, d50/d78/d106, d209/d237/d265<br>➢ Ex vivo B cell ELISpot (G1, G2, G3): Time points tested: d1, d11, d29/d57/d85, d36/d64/d92<br>➢ Bulk and single cell (10x) RNA sequencing (G1, G2, G3): Time points tested: d1, d11, d36/64/d92																
**PRISM EBL2001 (UK) Cohort 2 follow up**	Trial design/ Sample timepoints	G1															4 yrs	4.5 yrs
		G2															4 yrs	4.5 yrs
		G3															4 yrs	4.5 yrs
	Exploratory immunology	➢ BMEM ELISpot (G1, G2, G3): Time points tested: 4 years, 4.5 years post MVA-BN-Filo (dose 2)<br>➢ n=6 participants per group (paired samples at V1 an V2)																
**EBL2002 (Kenya, Burkina Faso, Uganda)**	Trial design/ Sample timepoints	G1						G1	d29					d50	d209	d365		
		G2	d1					G2	d57					d78	d237	d365		
		G3						G3	d85					d106	d265	d365		
	Exploratory immunology	➢ BMEM ELISpot (G1+G2 only): Time points tested: d1, d29/d57/d85, d50/d78/d106, d209/d237/d265, d365<br>➢ Only samples from participants in G1 (n=8) and G2 (n=11) were available from the Janssen Vaccines Central Biobank Storage on request under ethical approval from Medical Sciences Interdivisional Research Ethics Committee, University of Oxford, CUREC Approval reference R73519/RE001.																

Supplementary figure 1: Overview of Clinical trials and samples available for testing (cohort =recruitment cohort, Group = vaccination schedule based on day of MVA-BN-Filo administration): EBL2001 (UK) Cohort 1 (Group 1, n=8; Group 2, n=9; Group 3, n=9) samples available to determine the peak plasma cell time point by ex vivo, anti-EBOV-GP-IgG ELISpot; Plasma cell phenotyping and circulatory T follicular (cTfh) phenotyping by flow cytometry. EBL2001 (UK) Cohort 2: samples available for anti-EBOV-GP-IgG BMEM ELISpot (Group 1, n=15; Group 2, n=15; Group 3, n=18); B cell subset phenotyping by Flow cytometry (Group 1, n=10; Group 2, n=7; Group 3, n=11); ex vivo anti-EBOV-GP ELISpot (Group 1, n=35; Group 2, n=31, Group 3, n=32); Bulk and single cell (10X) RNA sequencing at d1, d11, d36/d64/d92. PRISM (UK) follow up study from EBL2001 cohort 2 with (n=6 participants available from vaccine Group 1, Group 2 and Group3) for anti-EBOV-GP-IgG BMEM ELISpot at V1 (4 years post dose 2) and V2 (4.5 years post dose 2). EBL2002 (Kenya, KY, Burkina Faso, BF and Uganda, UG) with PBMCs available from Group 1 (n=8) and Group 2(n=11) for anti-EBOV-GP IgG BMEM ELISpot (no samples available for Group 3).

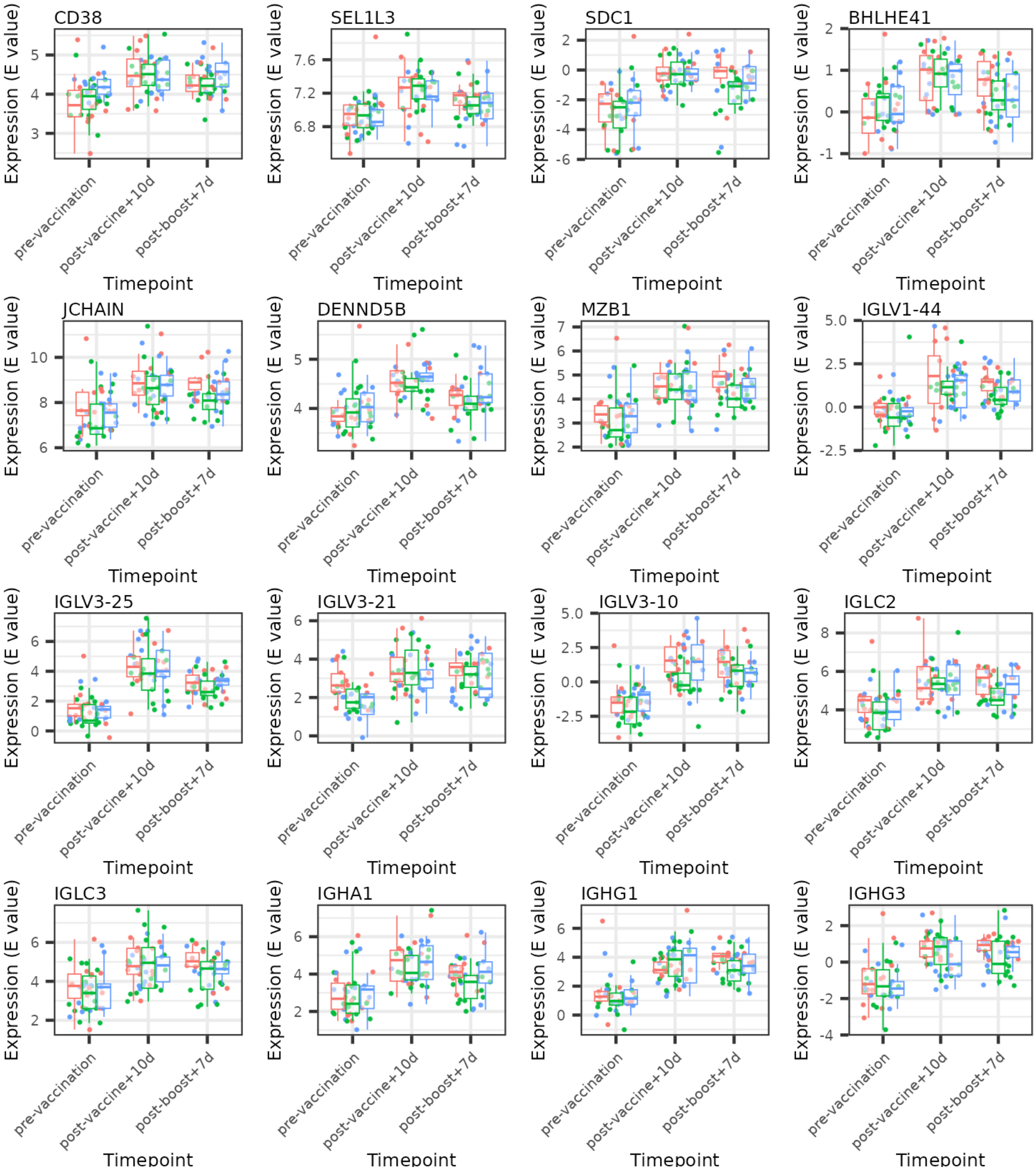

Supplementary figure 2: The 16 genes that were differentially expressed (FDR <0.05) 10 days after the first dose of vaccine and 7 days after the second vaccine.

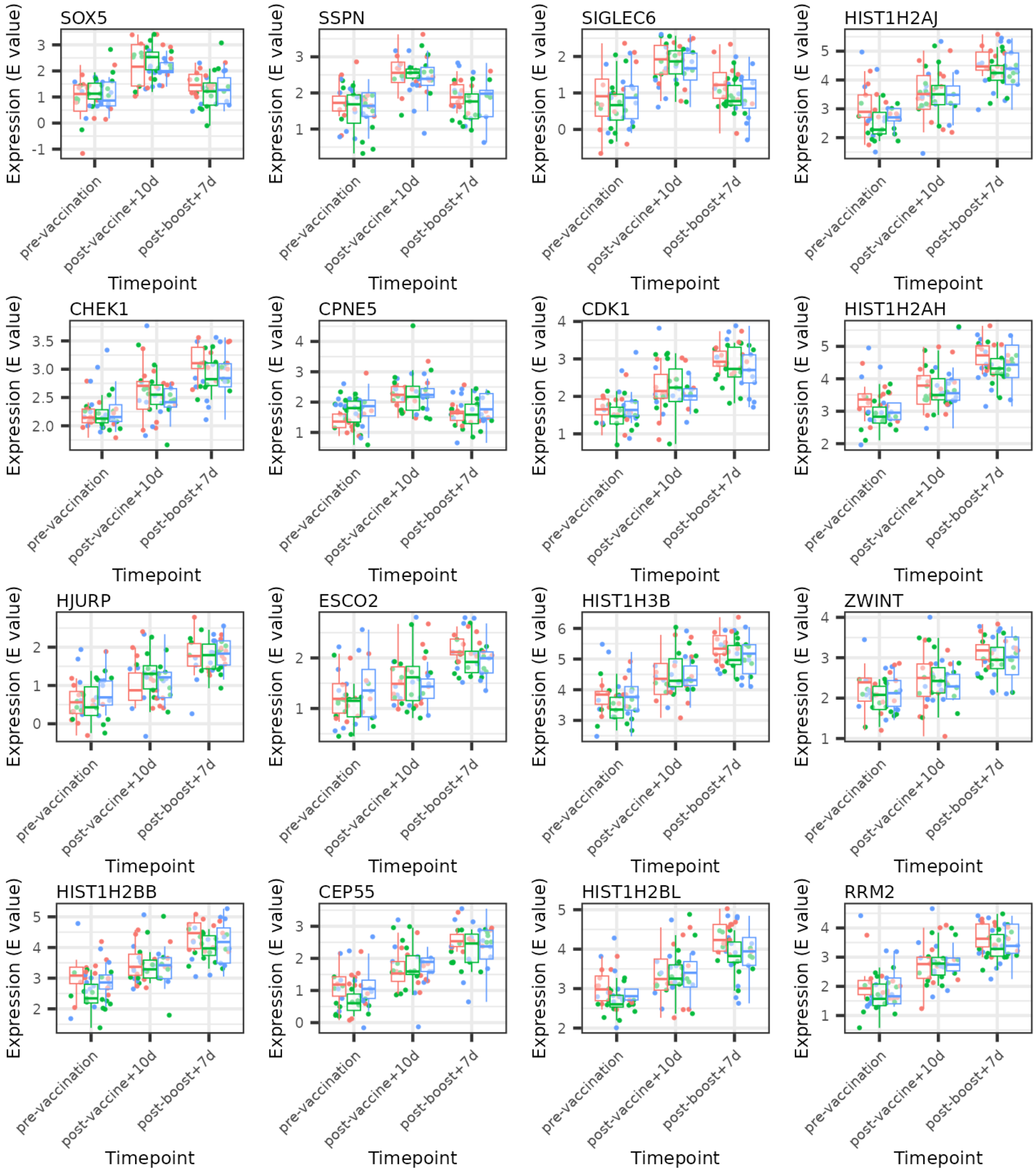

Supplementary figure 3: The top 16 differentially expressed gene (FDR <0.05) comparing 10 days after the first dose with 7 days after the second vaccine.

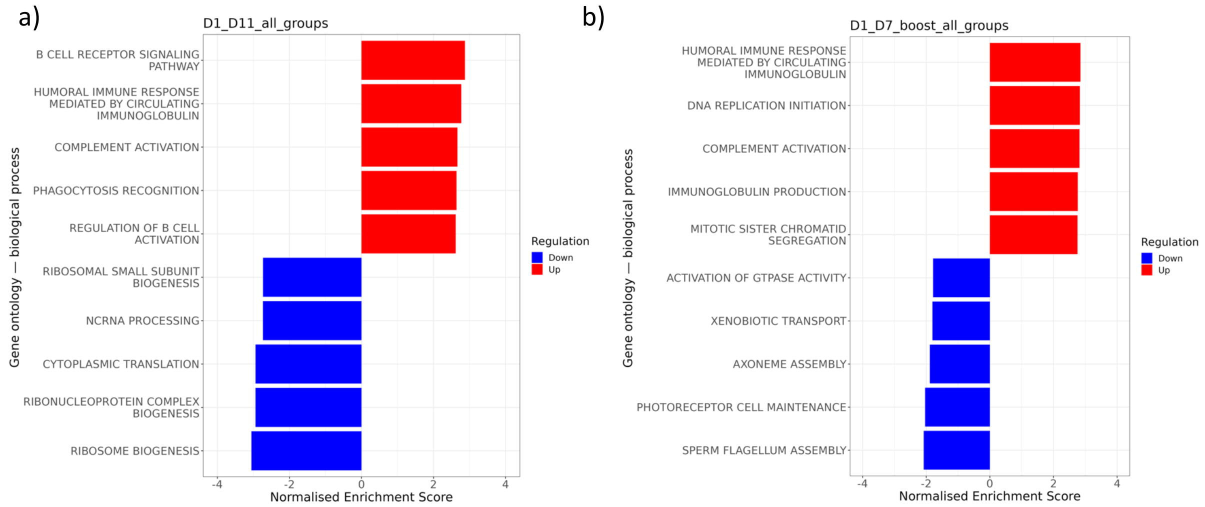

Supplementary figure 4: Top 5 upregulated and downregulated pathways from Gene set enrichment analysis (GSEA) 10 days (D11) after Ad26.ZEBOV (dose 1) compared with baseline (D1). Top 5 upregulated and downregulated pathways from Gene set enrichment analysis (GSEA) 7 days after MVA-BN-Filo (dose 2) compared with baseline (D1).

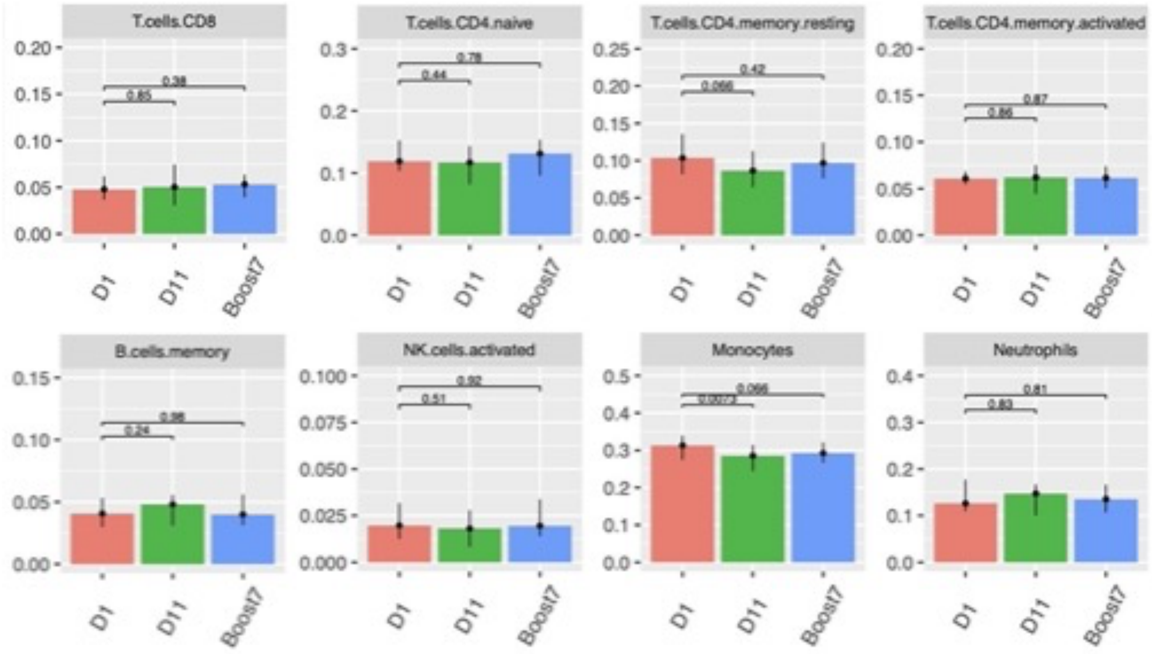

Supplementary figure 5: Plotted are the CIBERSORTx for various cell fractions from whole blood RNA-sequencing data, with median and interquartile range. P-values were determined from a two-sample Wilcoxon rank sum test.

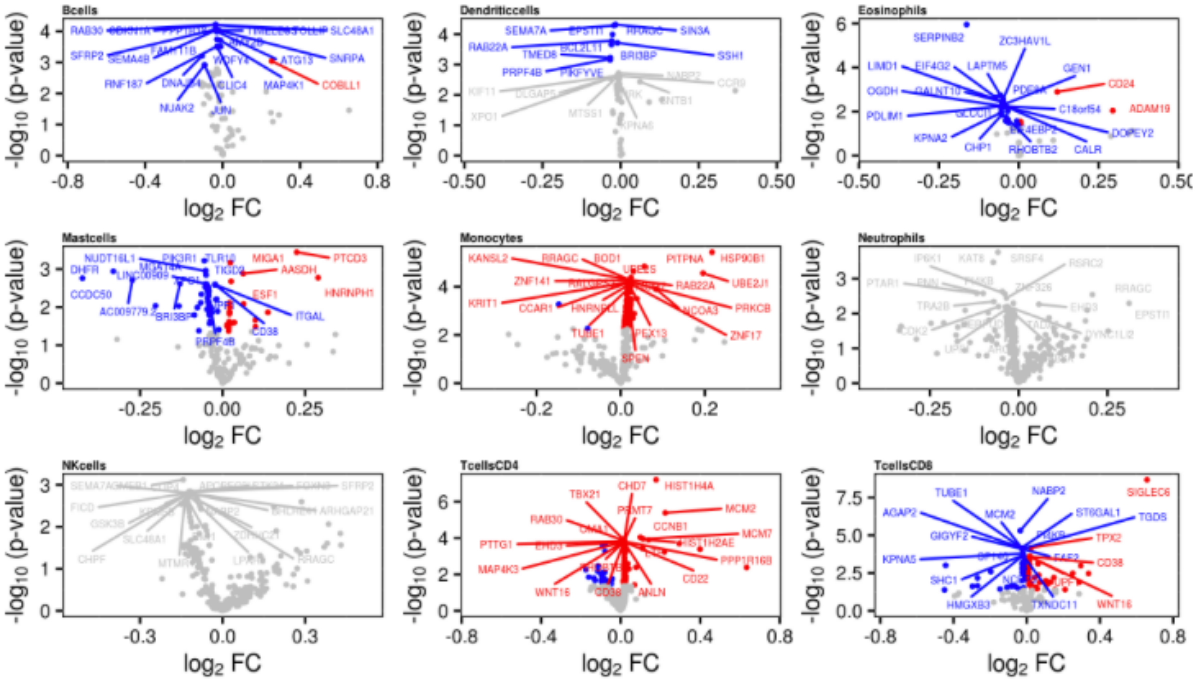

Supplementary figure 6: Volcano plot derived from imputed cell subset gene expression using CIBERSORTx on the 1000 most differentially expressed genes at day 11, highlighting differentially expressed genes (DEGs, false discovery rate [FDR] < 0.05; red upregulated and blue downregulated) day 11 after first dose of Ad26.ZEBOV vaccine (all groups) compared with pre-vaccination (n = 40).

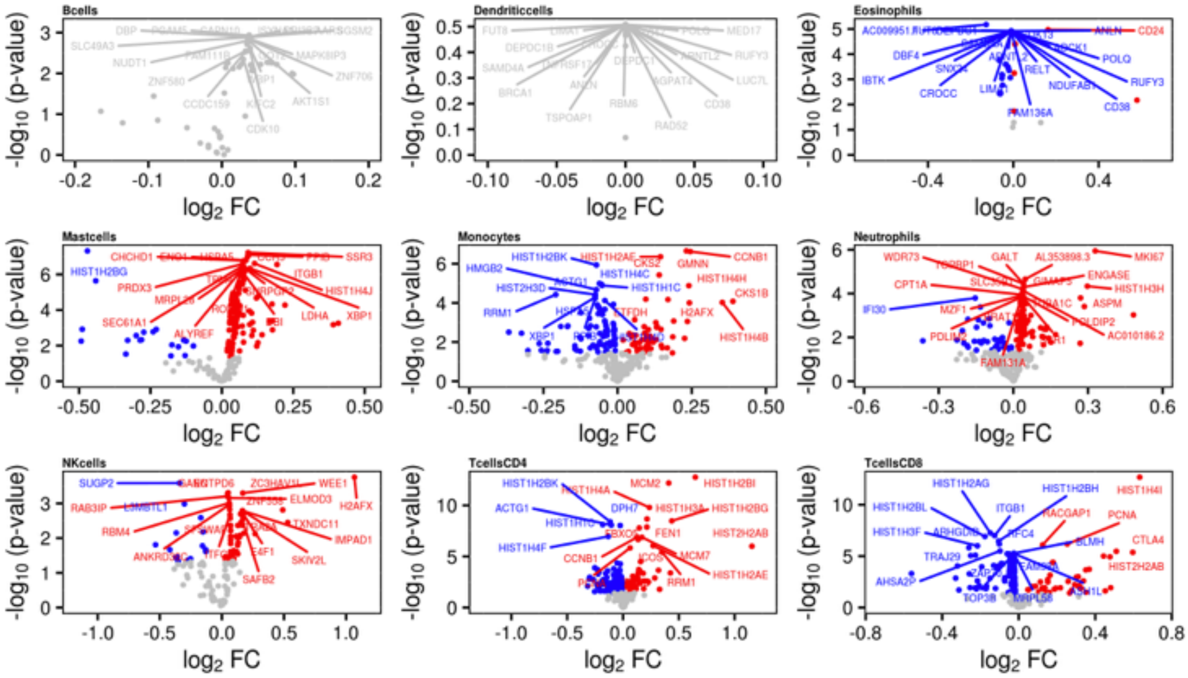

Supplementary figure 7: Volcano plot derived from imputed cell subset gene expression using CIBERSORTx on the 1000 most differentially expressed genes at day 7, highlighting differentially expressed genes (DEGs, false discovery rate [FDR] < 0.05; red upregulated and blue downregulated) day 7 after second vaccine dose (all groups) compared with pre-vaccination (n = 28).

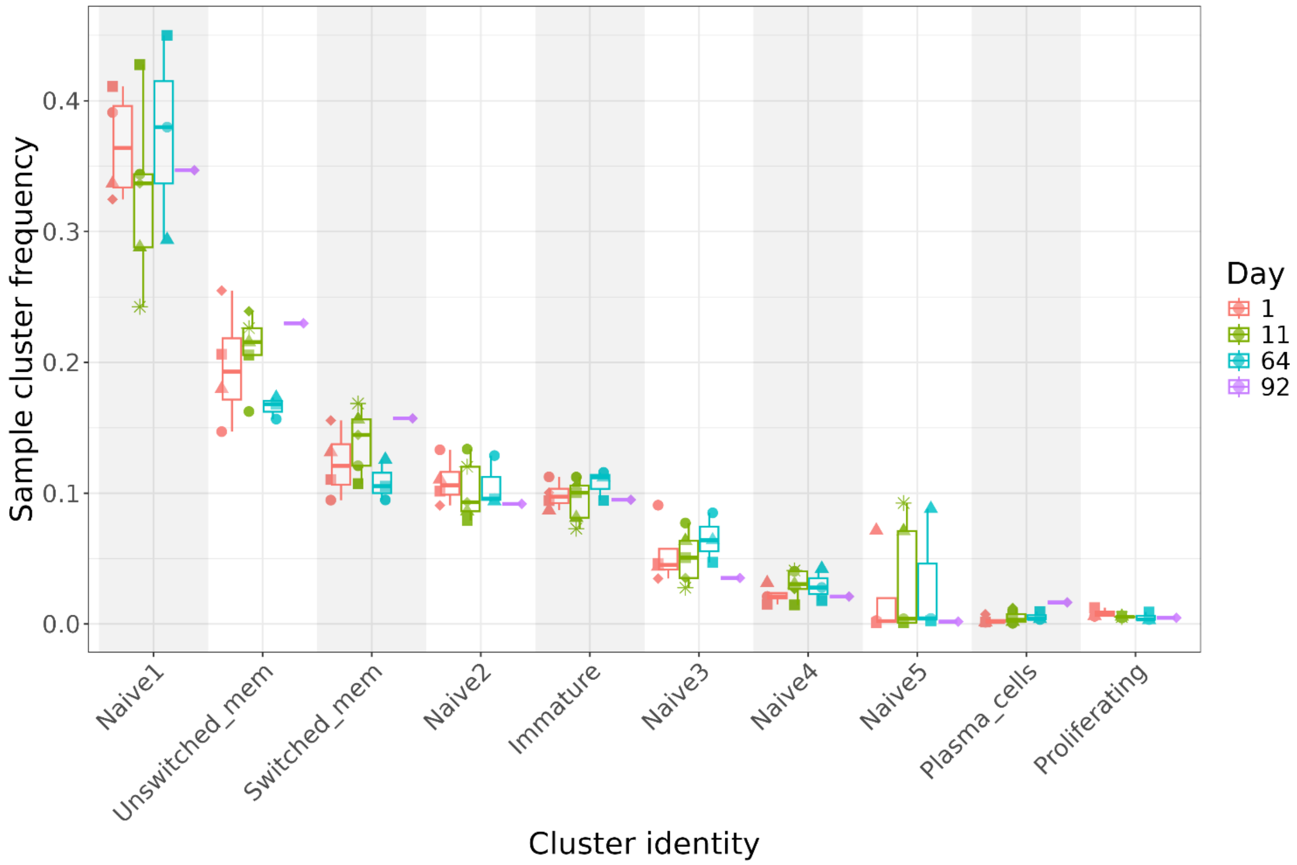

Supplementary figure 8: B Cell cluster frequencies at the study time points.

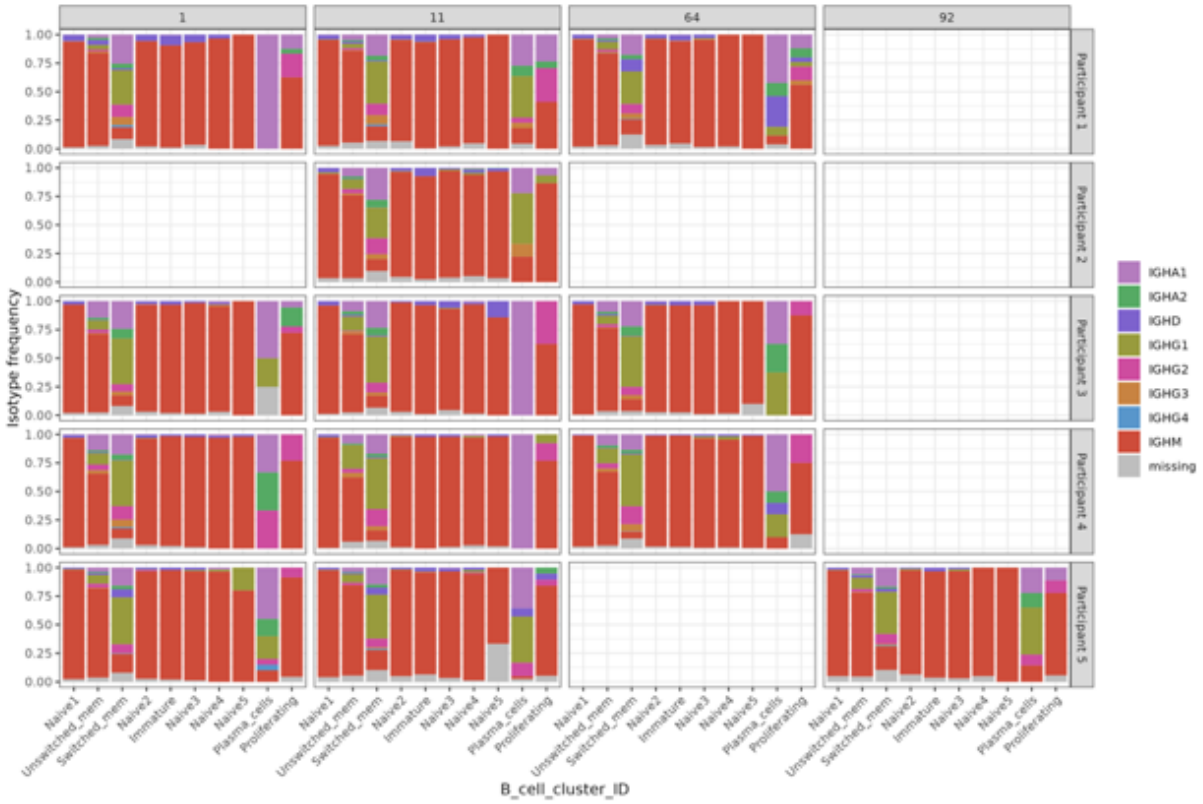

Supplementary figure 9: Immunoglobulin subclass frequency in single cell RNA-seq B cell clusters at the study time points.

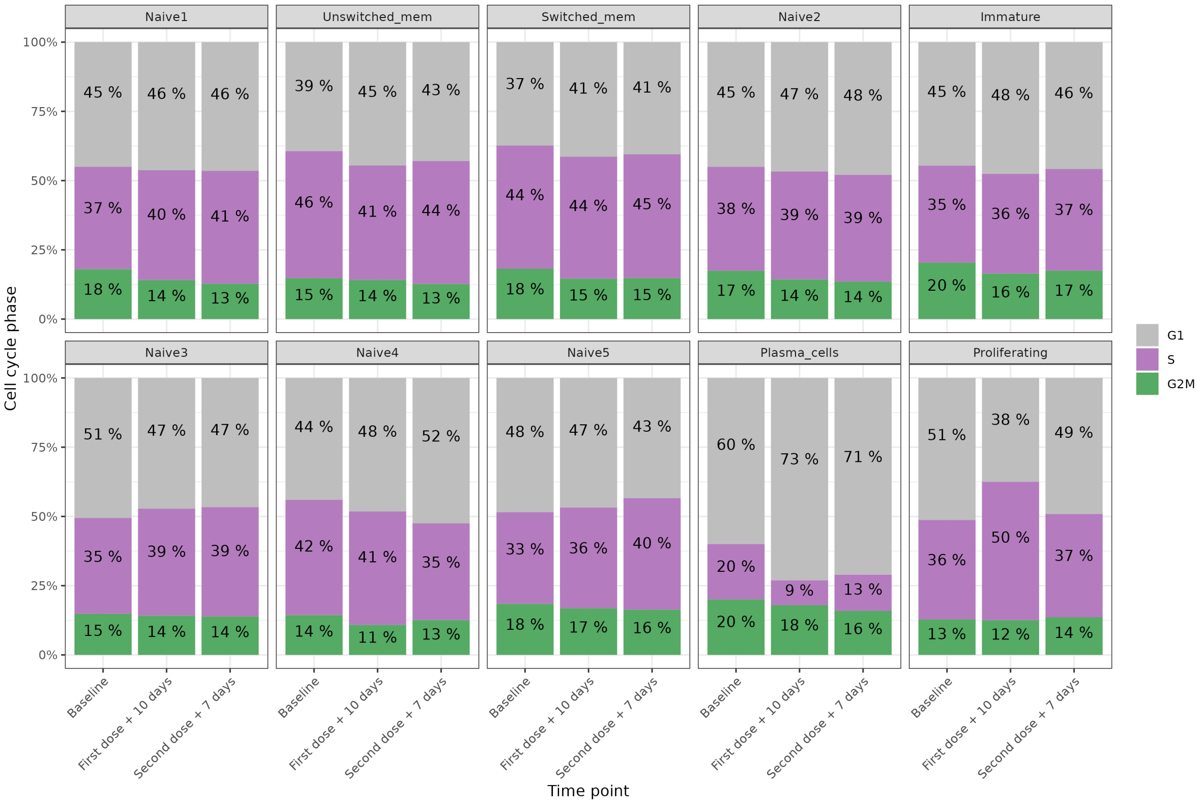

Supplementary figure 10: B cell subset cell cycle phase proportions — inferred from the expression of G2/M and S phase gene markers in single cell RNA-sequencing data— at the study time points.

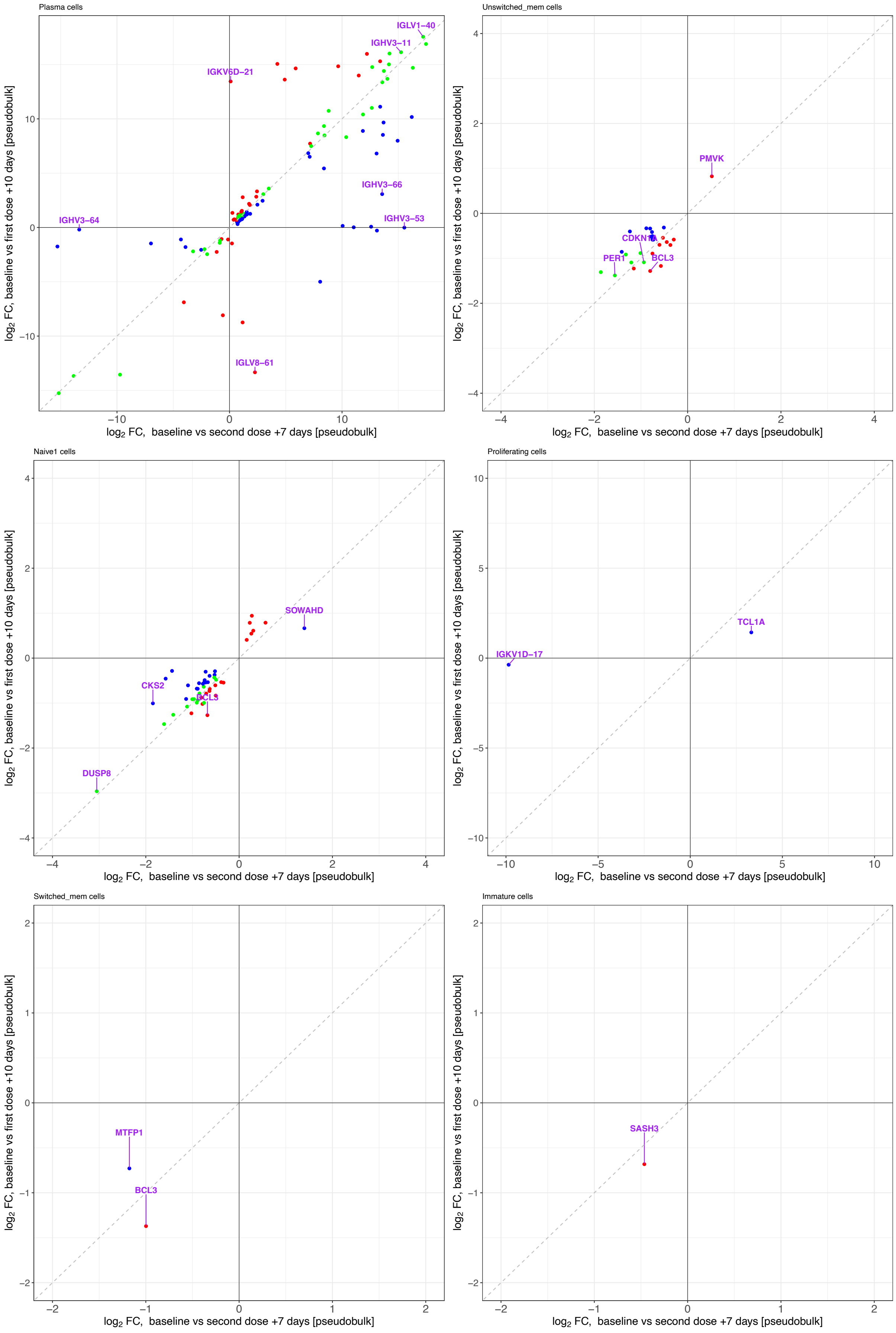
Plasma cells
IGLV1–40
IGHV3–11
IGKV6D–21
IGHV3–66
IGHV3–64
IGHV3–53
IGLV8–61
Unswitched_mem cells
PMVK
CDKN1A
PER1
BCL3
Naive1 cells
SOWAHD
CKS2
BCL3
DUSP8
Proliferating cells
TCL1A
IGKV1D–17
Switched_mem cells
MTFP1
BCL3
Immature cells
SASH3
log2 FC, baseline vs first dose +10 days [pseudobulk]
log2 FC, baseline vs second dose +7 days [pseudobulk]

Supplementary figure 11: Agreement plots. Red = differentially expressed genes after first vaccine dose, blue = differentially expressed genes after second vaccine dose, green = differentially regulated REACTOME pathways after both vaccine dose.

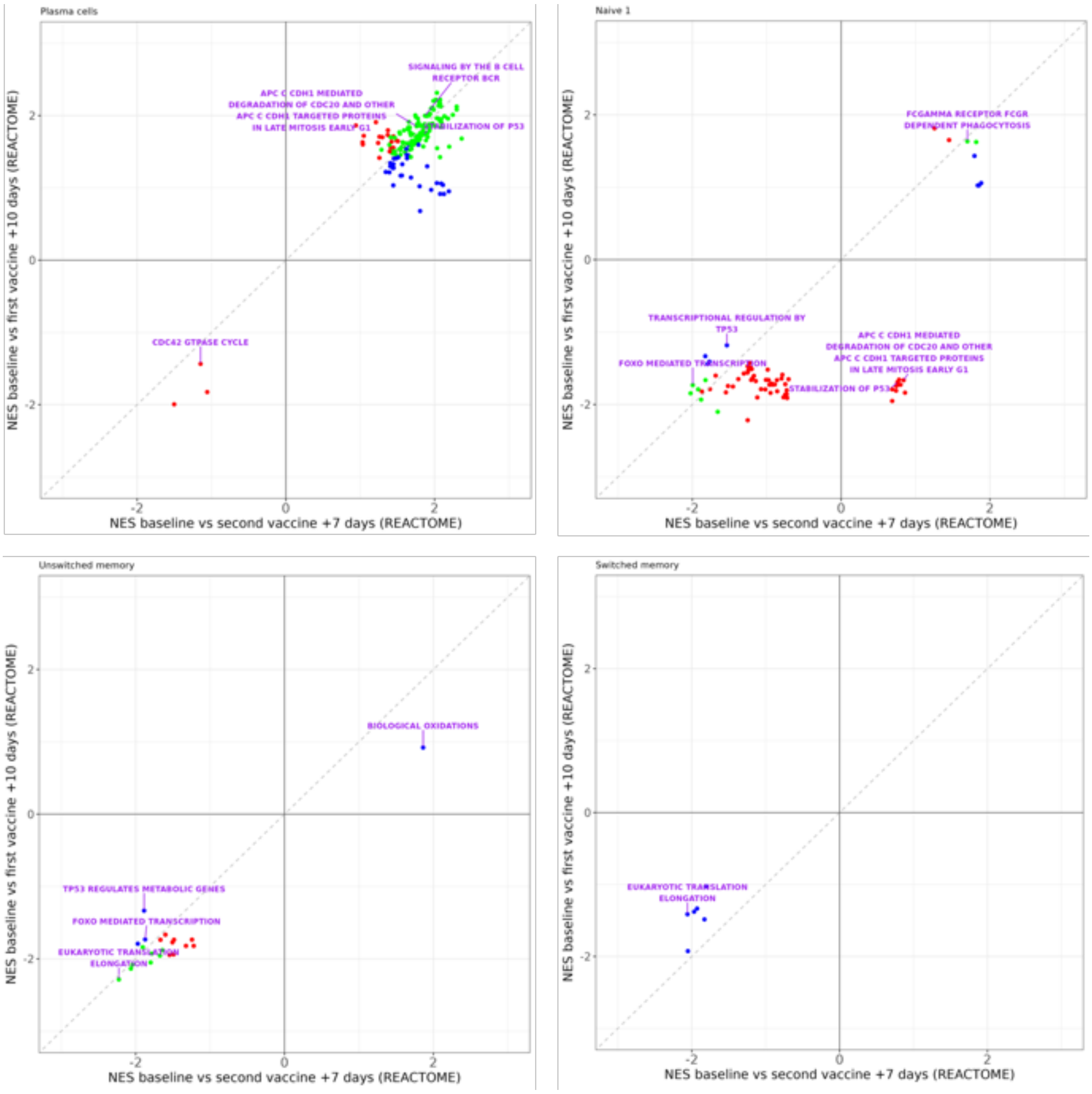

Supplementary figure 12: Agreement plots. Red = differentially regulated REACTOME pathways after first vaccine dose, blue = differentially regulated REACTOME pathways after second vaccine dose, green = differentially regulated REACTOME pathways after both vaccine dose.

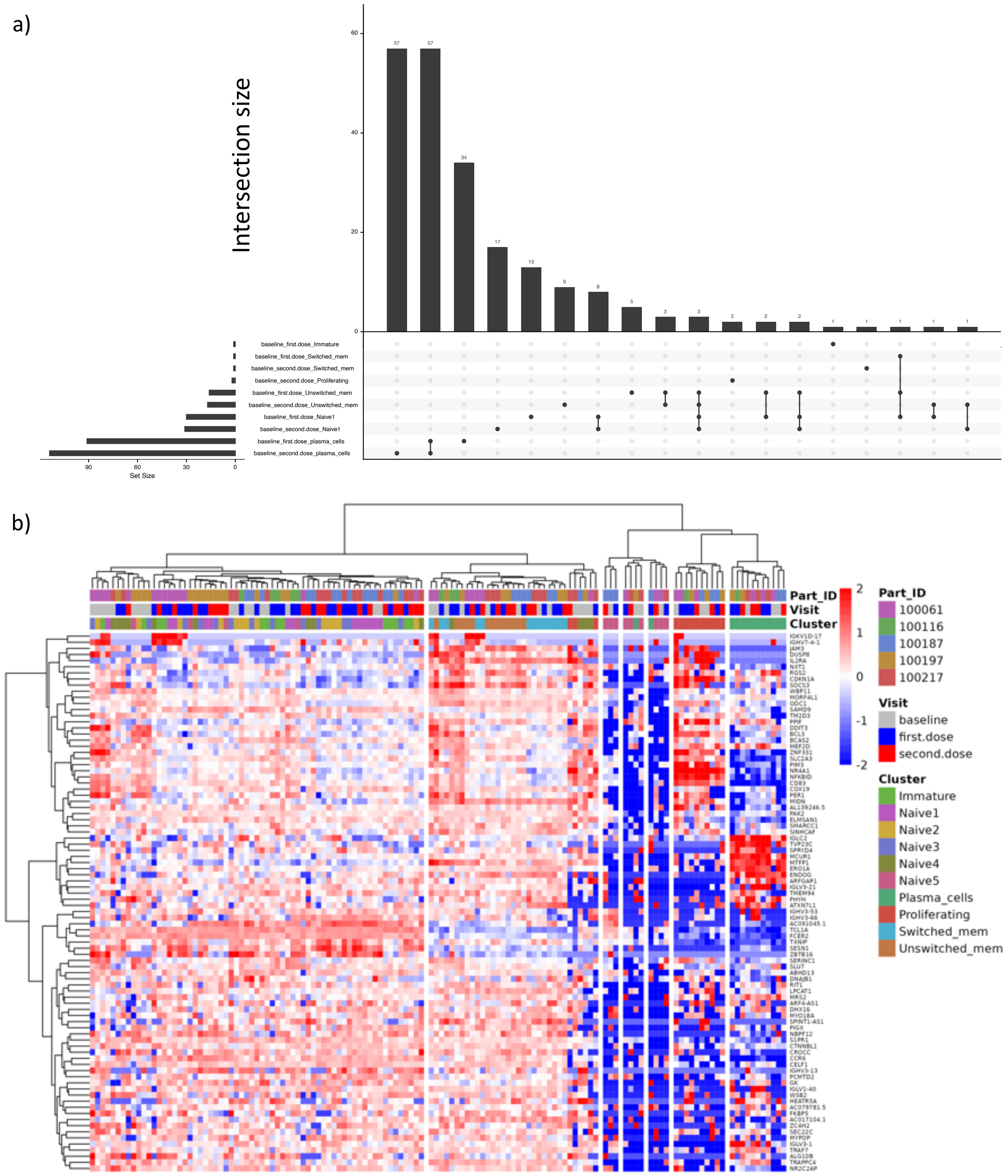

Supplementary figure 13: a) UpSet plot of differentially expressed genes (DEGs, false discovery rate [FDR] < 0.01) in pseudobulk data at study time points. b) Heatmap of top 5 upregulated genes in pseudobulk analysis of each cell cluster post-vaccination compared with pre-vaccination.

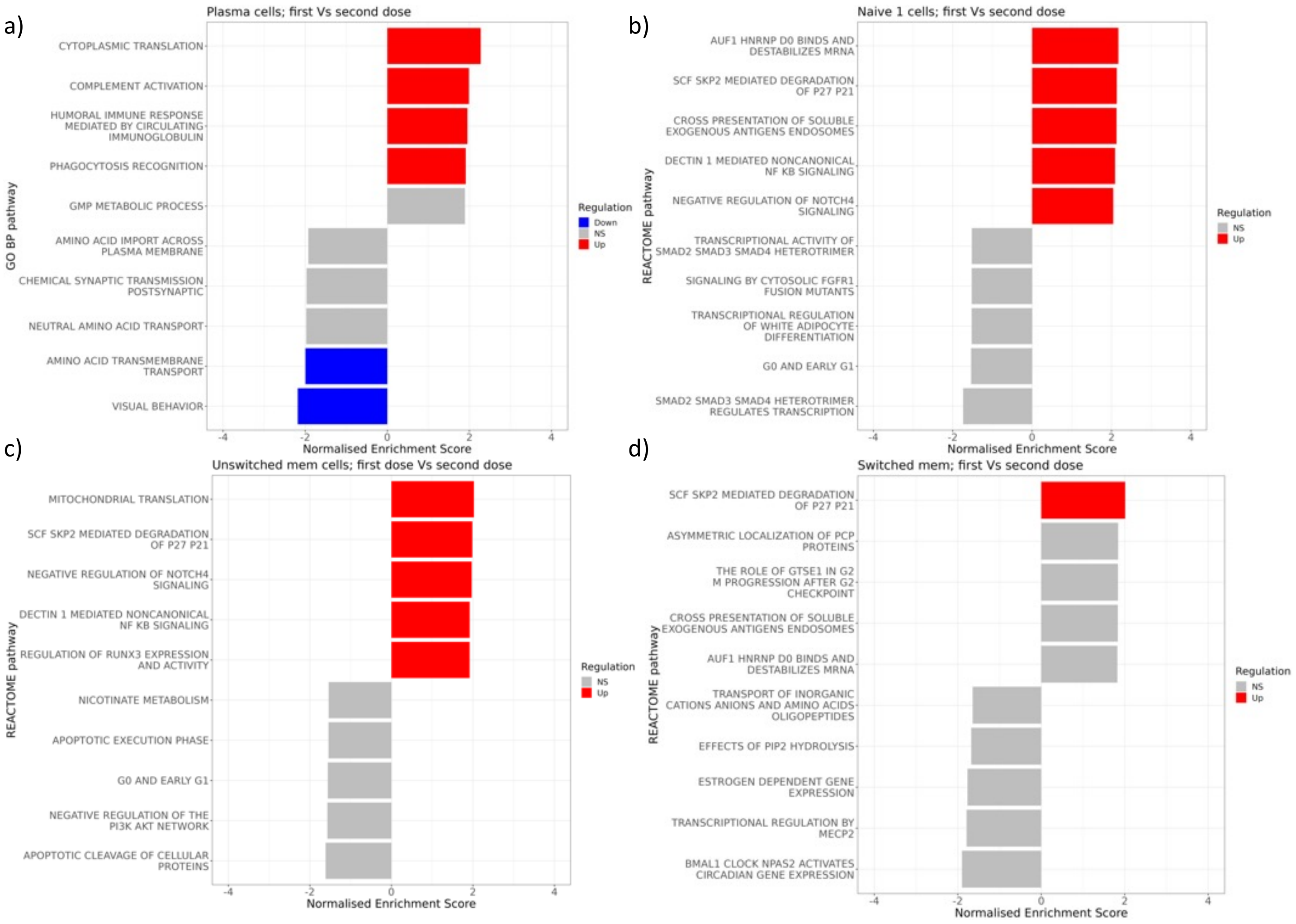

Supplementary figure 14:Top 5 upregulated and downregulated pathways from Gene set enrichment analysis (GSEA) days after MVA-BN-Filo (dose 2) compared with 10 days (D11) after Ad26.ZEBOV (dose 1) in different pseudobulk cell populations, a) plasma cells, b) naïve 1 cells, c) unswitched memory and d) switched memory B cells.

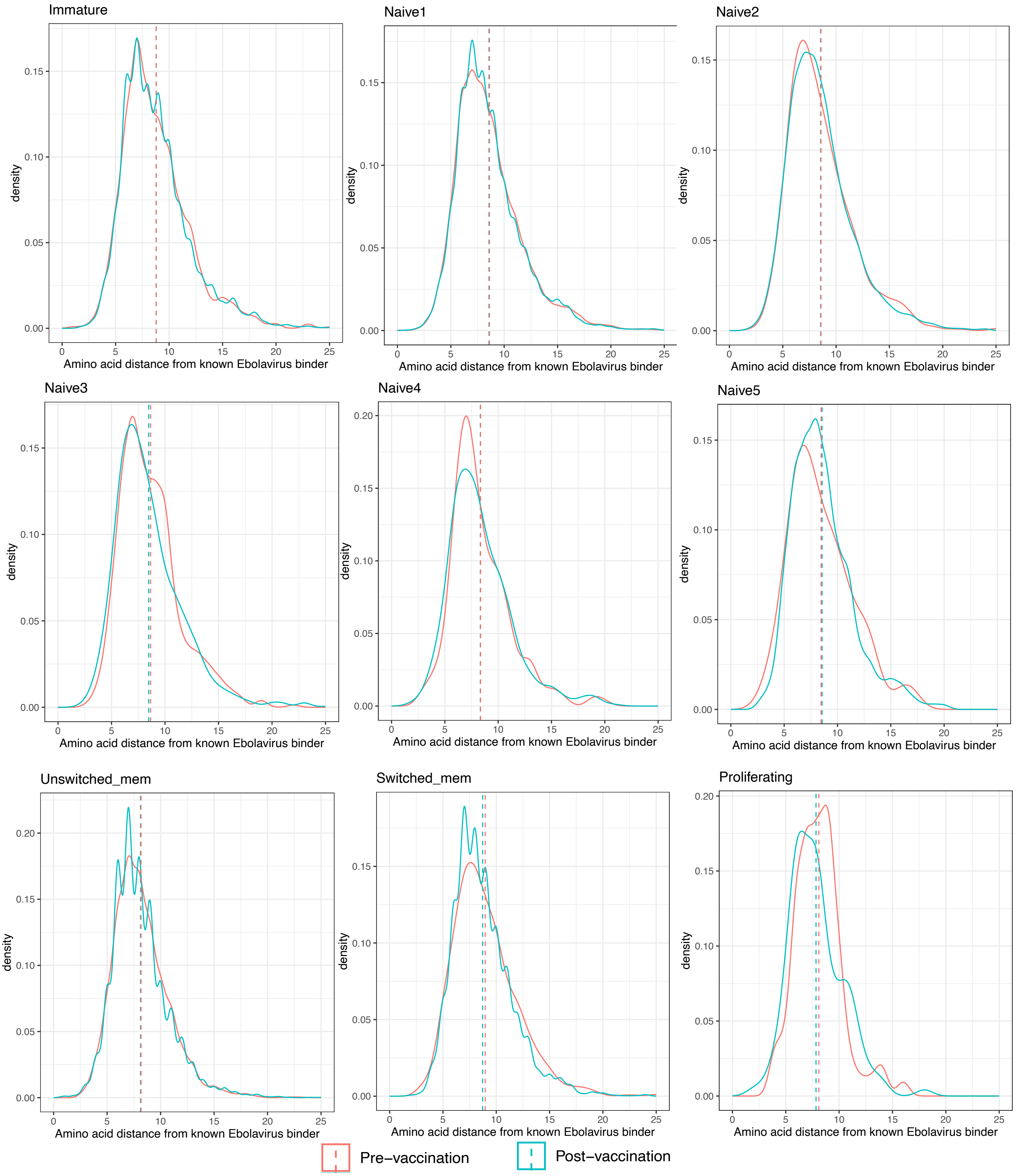

Supplementary figure 15: Hamming distance of CDRH3 amino acid sequences from different B cell subsets to known Ebola glycoprotein binding antibody sequence

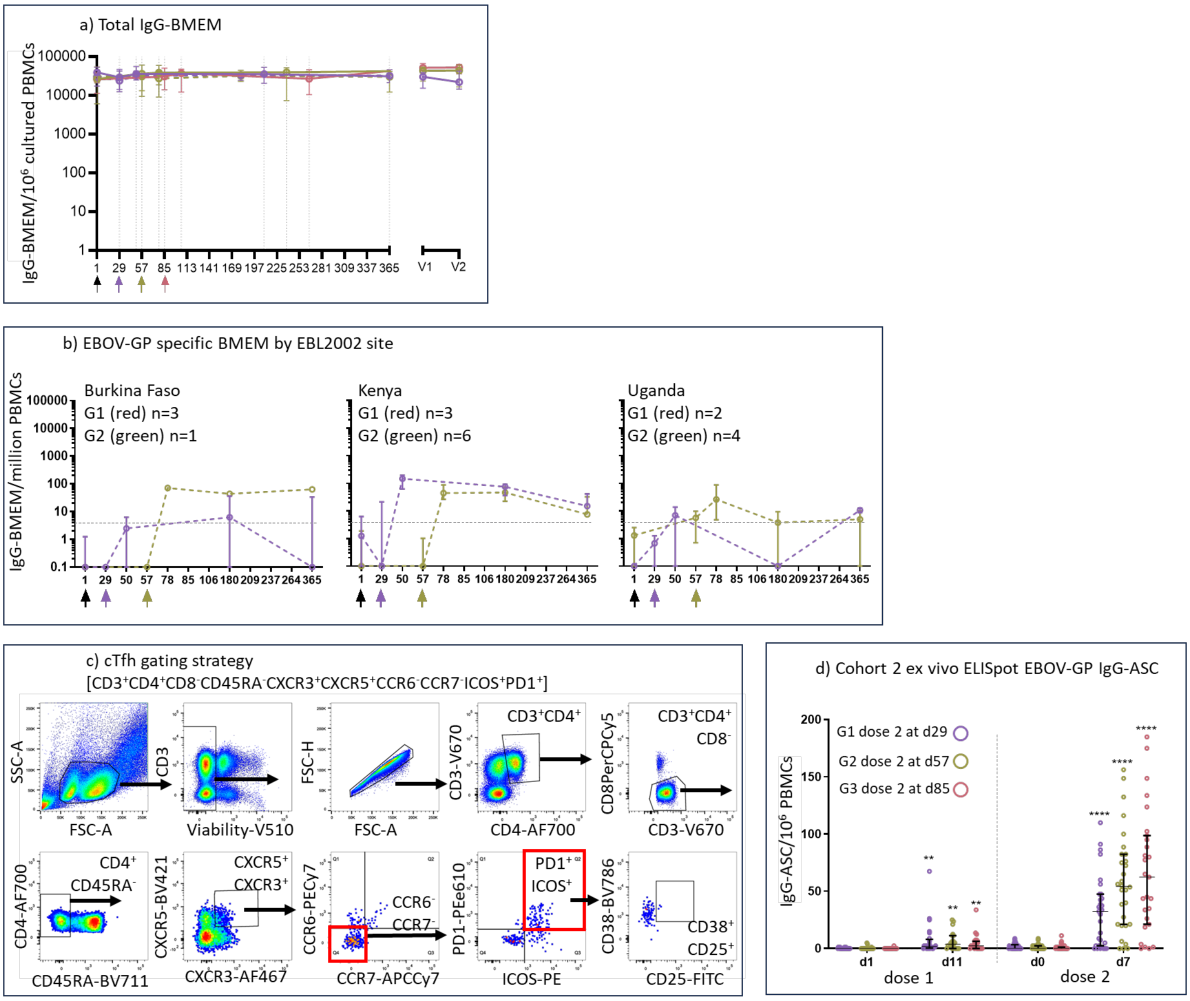

Supplementary figure 16: Total IgG-BMEM, EBOV-GP specific IgG-BMEM by site, cTfh gating and EBOV-GP ASC

*a) Total-IgG-BMEM for all studies: Vaccine Group 1 (purple), Group 2 (olive) and Group 3 (coral, UK only). Median (+/- IQR) are shown as solid lines for EBL2001 and PRISM, while dotted lines are shown for EBL2002. b) EBOV-GP specific IgG-BMEM from participants in EBL2002 split by trial country (Burkina Faso, Kenya and Uganda) with number of participants shown on each site graph, data presented as in (a). c) cTfh gating strategy for EBL2001 (graphical representation in Figure 1g). d) Frequency of ex vivo EBOV-GP specific IgG-ASC from participants in EBL2001 at study day (d11) following Ad26.ZEBOV (dose 1) and d7 post MVA-BN-Filo (dose 2) given at d29 (G1, purple); d57 (G2, olive) or d85 (G3, coral). ANOVA with Freidman test ** p=<0.01 above d1, ****p=<0.0001 above d0 (pre dose 2).*

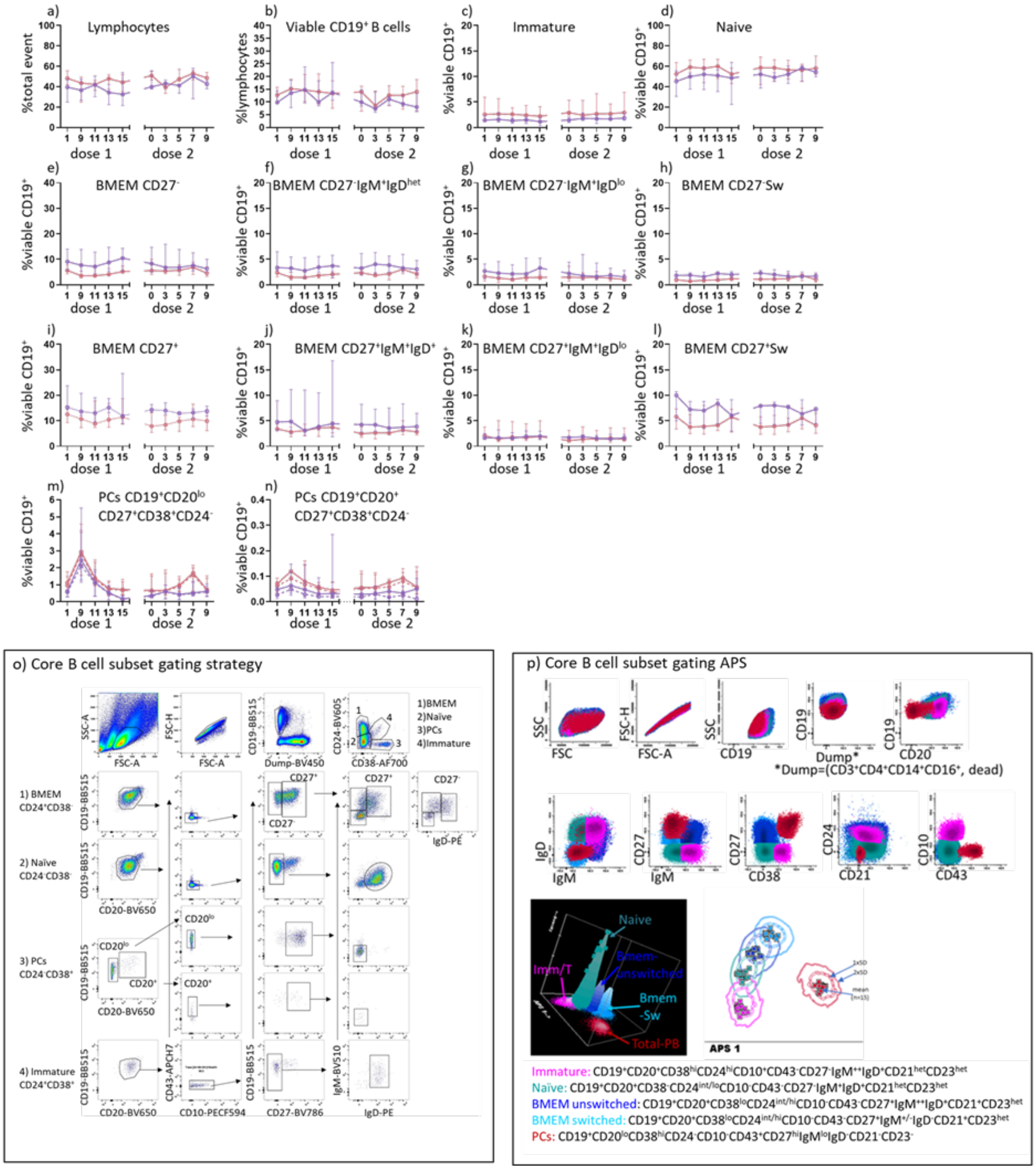

Supplementary figure 17: a-n) Frequency of core B cell subsets following Ad26.ZEBOV (dose 1, d1-d15) and MVA-BN-Filo (dose 2, d0-d9) given at either d29 post dose 1 (purple, Group 1) or d85 (Group 3, coral), data are expressed as %viable CD19+ B cells and graphs show the Median (+/-IQR). o) Core B cell subset gating using Cohort 1 panel A antibody cocktail (Supplementary Table 11, supplementary methods), and analysed in Flow Jo v10.1.1). p) APS plot, with contributory gating, showing the relative sizes of the core B cell subsets, and reproducibility of the gating capture for all samples (squares, +2xSD contours), analysed in InfinicytTM V1.8.

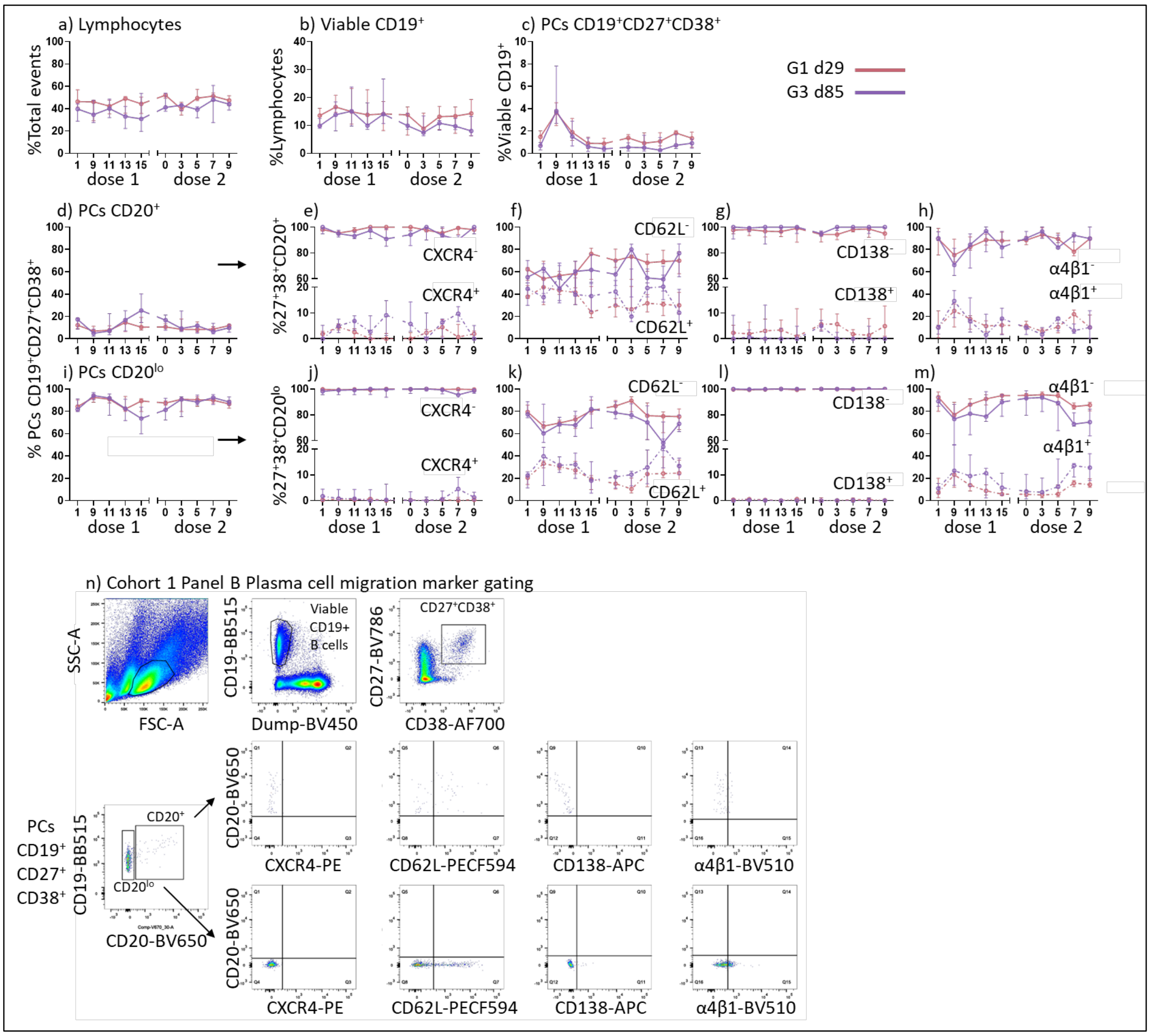

Supplementary figure 18: a-m) Frequency of migratory plasma cell subsets following Ad26.ZEBOV (dose 1, d1-d15) and MVA-BN-Filo (dose 2, d0-d9) given at either d29 post dose 1 (purple, Group 1) or d85 (Group 3, coral). Total plasma cells (PCs) data are expressed as %viable CD19+ B cells (c). Data for plasma cell subsets are expressed as %PCs CD19⁺CD27⁺CD38⁺CD20⁺ (e-h) or %PCs CD19⁺CD27⁺CD38⁺CD20^lo (j-m). Graphs show the Median (+/-IQR). n) Migratory plasma cell subset gating using Cohort 1 panel B antibody cocktail (Supplementary Table 11, supplementary methods) and analysed in Flow Jo v10.1.1).

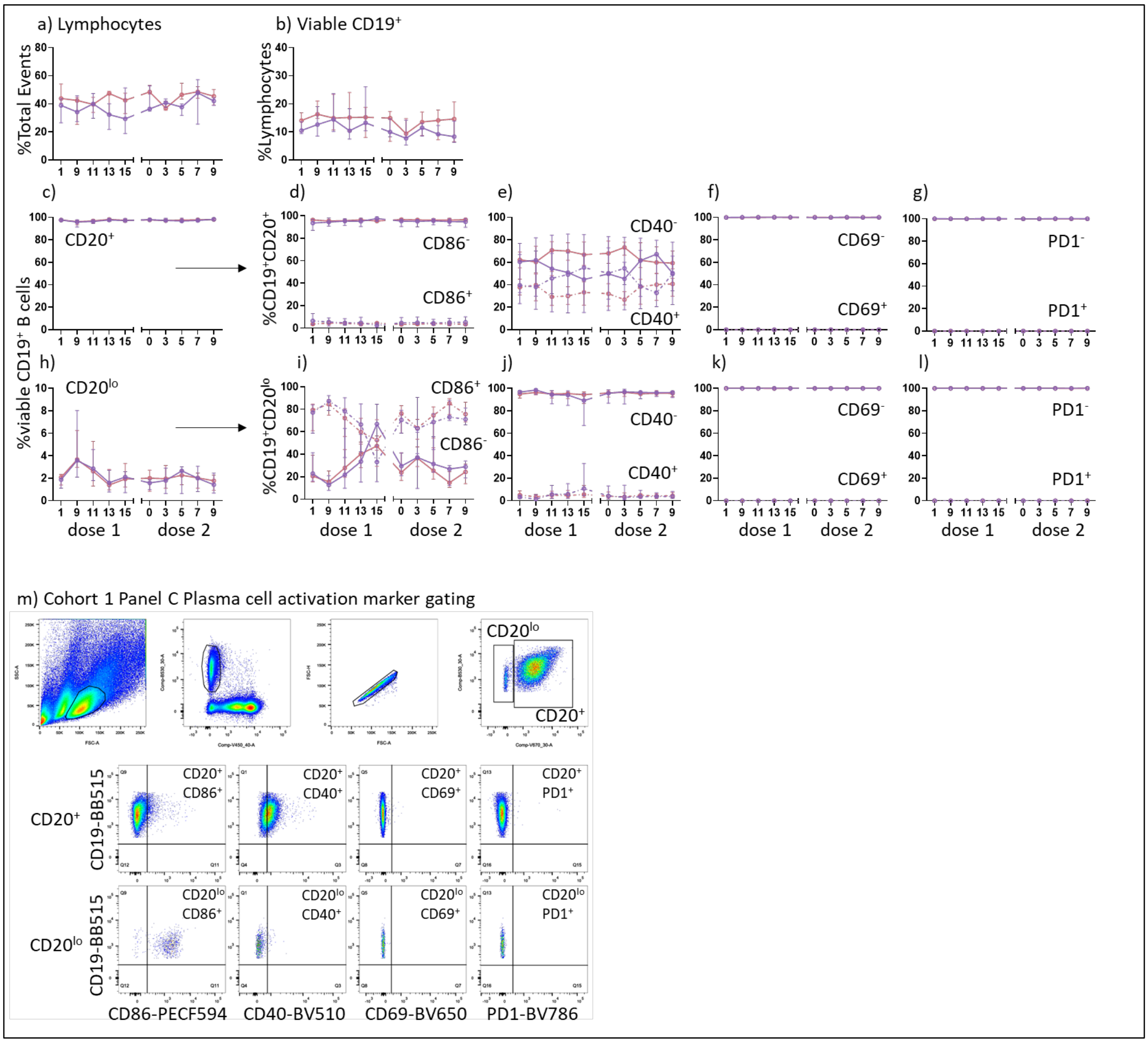

Supplementary figure 19: a-m) Frequency of activated plasma cell subsets, expressing CD40, CD69, CD86 or PD1 following Ad26.ZEBOV (dose 1, d1-d15) and MVA-BN-Filo (dose 2, d0-d9) given at either d29 post dose 1 (purple, Group 1) or d85 (Group 3, coral), data are expressed as either %viable CD19+ B cells (c+h) or CD19^{+}CD20^{+} (d-g)or CD20^{lo} (i-l). Graphs show the Median (+/-IQR). m) Gating using Cohort 1 panel C antibody cocktail (Supplementary Table 11, Supplementary Methods) and analysed in Flow Jo v10.1.1).

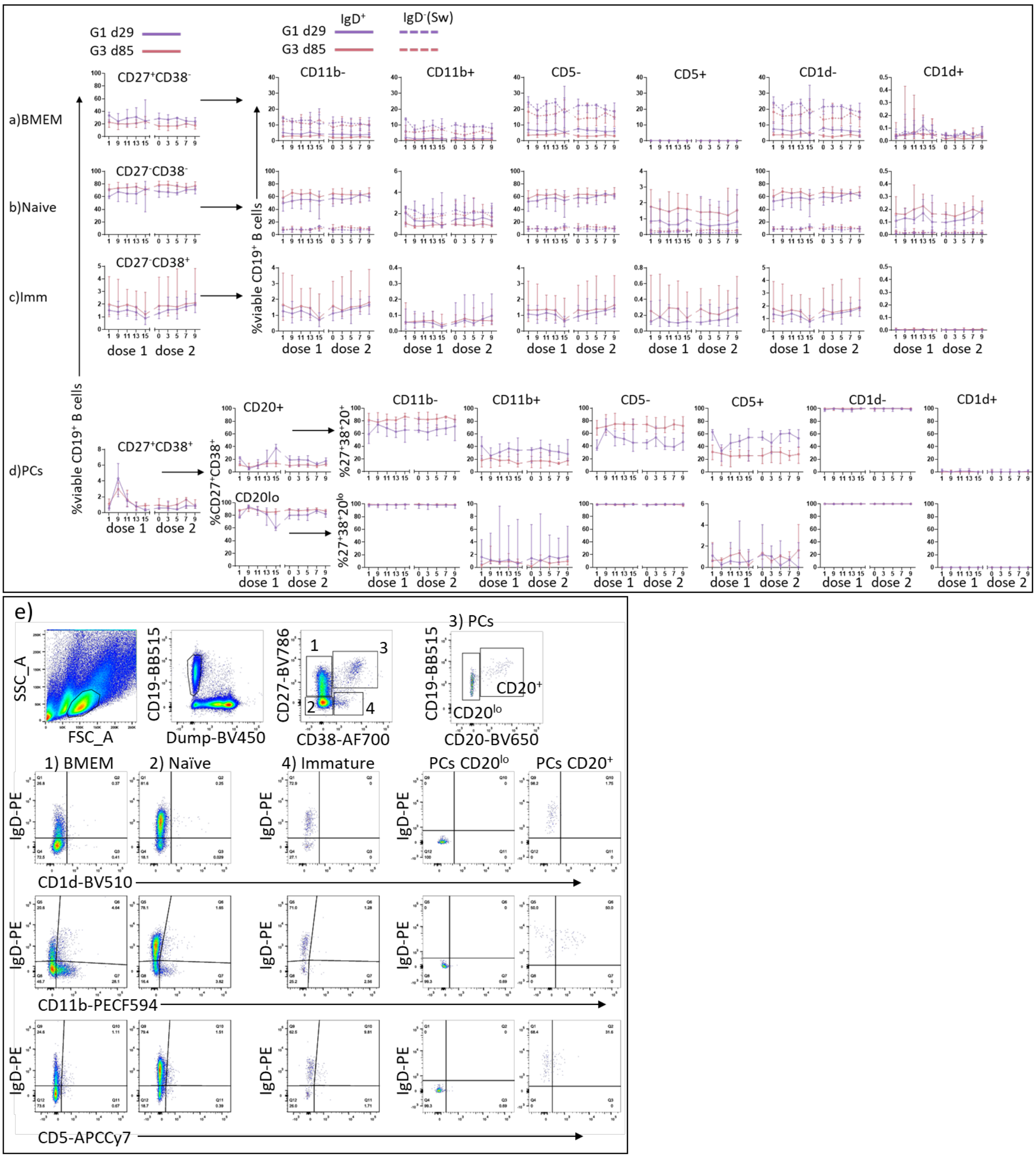

Supplementary figure 20: Expression of CD11b, CD5, CD1d by core B cell subsets to identify the appearance of Innate like B cells following Ad26.ZEBOV (dose 1, d1-d15) and MVA-BN-Filo (dose 2, d0-d9) given at either d29 post dose 1 (purple, Group 1) or d85 (Group 3, coral). For frequency within a) BMEM, b) Naive, and c) Immature (Imm) data are expressed as %viable CD19+ B cells. For d) plasma cells (PC), which are either CD20+ or CD20lo data are expressed as %CD19+CD27+CD38+. Graphs show the Median (+/-IQR). e) Gating using Cohort 1 panel D antibody cocktail (Supplementary Table 11, Supplementary Methods) and analysed in Flow Jo v10.1.1.

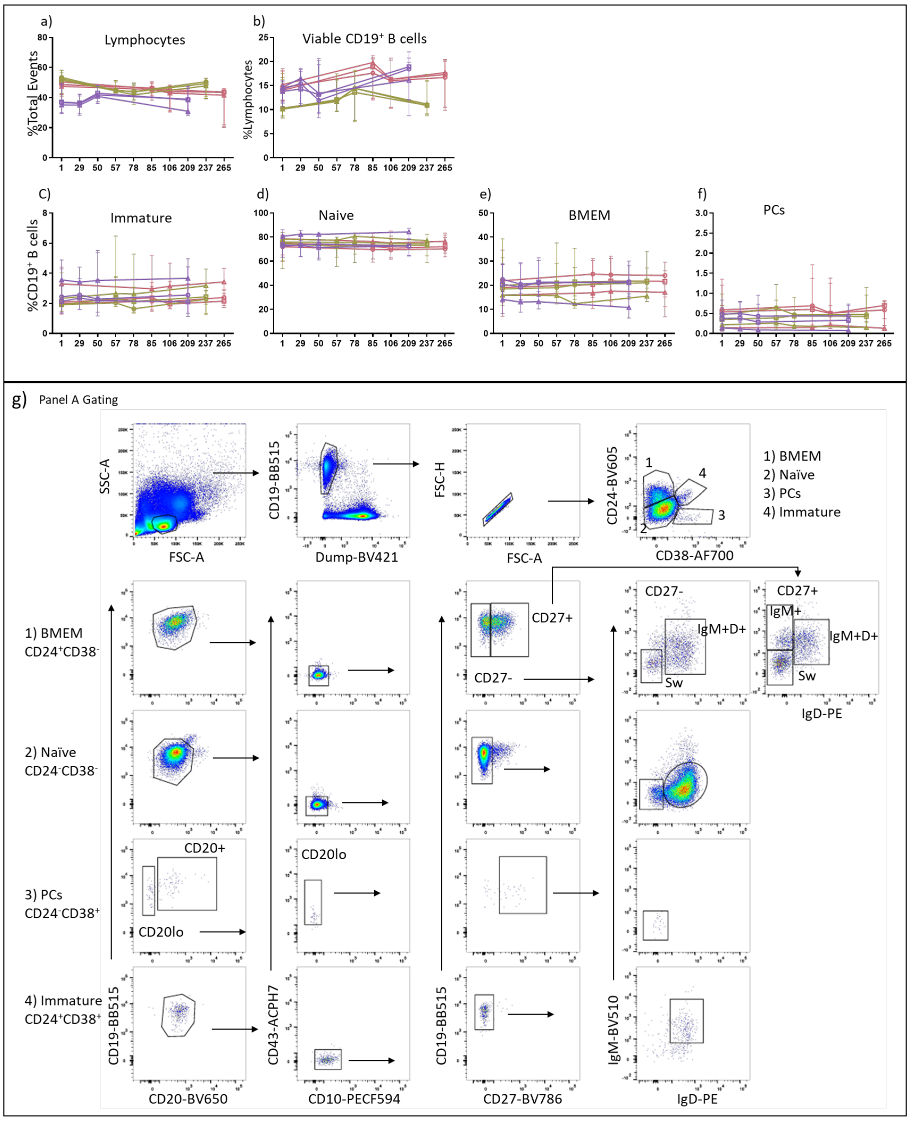

h)

Immature CD19+CD24+CD38+CD20+CD43-CD10+CD27-
- **Immature CD19+CD24+CD38+CD20+CD43-CD10+CD27-[M+D+]**

Naïve CD19+CD24-CD38-CD20+CD43-CD10-CD27-
- **Naïve CD19+CD24-CD38-CD20+CD43-CD10-CD27-[M+D+]**
- **Naïve CD19+CD24-CD38-CD20+CD43-CD10-CD27-[Sw]**

BMEM CD19+CD24+CD38-CD20+CD43-CD10-
- **BMEM CD19+CD24+CD38-CD20+CD43-CD10-CD27-**
  - **BMEM CD19+CD24+CD38-CD20+CD43-CD10-CD27-[M+D+]**
  - **BMEM CD19+CD24+CD38-CD20+CD43-CD10-CD27-[Sw]**
- **BMEM CD19+CD24+CD38-CD20+CD43-CD10-CD27+**
  - **BMEM CD19+CD24+CD38-CD20+CD43-CD10-CD27+[M+D+]**
  - **BMEM CD19+CD24+CD38-CD20+CD43-CD10-CD27+[M+Dlo]**
  - **BMEM CD19+CD24+CD38-CD20+CD43-CD10-CD27+[M-D+]**
  - **BMEM CD19+CD24+CD38-CD20+CD43-CD10-CD27+[Sw]**

PCs CD19+CD24-CD38+CD43+CD10-CD27+
- **PBs CD19+CD24-CD38+CD43+CD10-CD27+CD20+**
- **PBs CD19+CD24-CD38+CD43+CD10-CD27+CD20+[Sw]**
- **PBs CD19+CD24-CD38+CD43+CD10-CD27+CD20lo**
- **PBs CD19+CD24-CD38+CD43+CD10-CD27+CD20lo[Sw]**

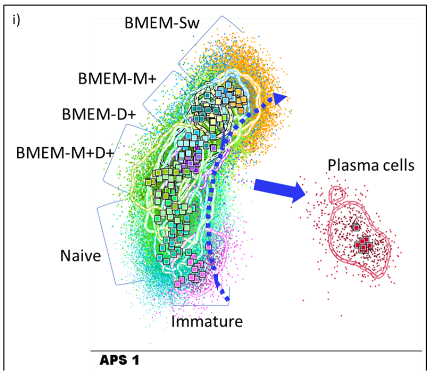

Supplementary figure 21: EBL2001 Cohort 2 Core B cell subset gating and comparison of frequencies between panels A+B+C. The kinetics of the populations are shown in the graphs (a-f) with Group 1 (purple), Group 2 (olive) and Group 3 (coral): a) Lymphocytes (% total events); b) viable CD19+ B cells (% Lymphocytes): c) Immature B cells, d) Naïve B cells, e) BMEM, f) PCs (all expressed as % viable CD19+ B cells). Triplicate lines show frequencies gated with each of the antibody panels used (A, B, C, Supplementary Table 12, Supplementary Methods). The gating strategy used for the core B cell subsets using antibody panel A is shown in (g) and the surface markers used to delineate the core B cells subsets (h) and analysed in Flow Jo v10.1.1. An APS based on this gating strategy is show in (i) with squares showing each sample (n=11) and contour lines showing 1xSD for each population, obtained using Infinicyt™

version 1.8. The dotted arrow shows the maturation direction with plasma cells derived from BMEM populations (Solid arrow).

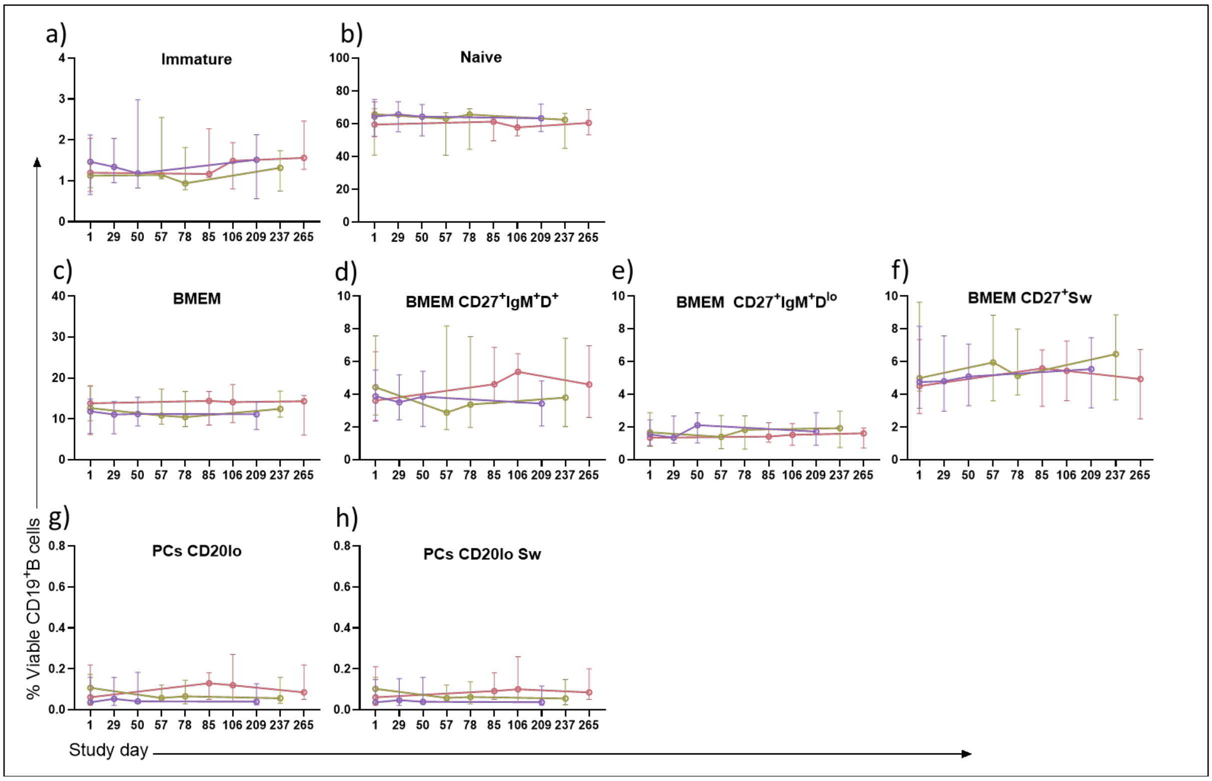

Supplementary figure 22: Core B cell subsets gated as shown in Supple. Fig. 21(i): Immature, Naïve, BMEM, BMEM M+D+, BMEM M+Dlo, BMEM Sw, PCs (CD20lo) and CD20lo Switched (Sw). Data are expressed as percentage of viable, CD19+ B cells and show median +/-IQR, for Group 1 (purple), Group 2 (olive) and Group 3 (coral).

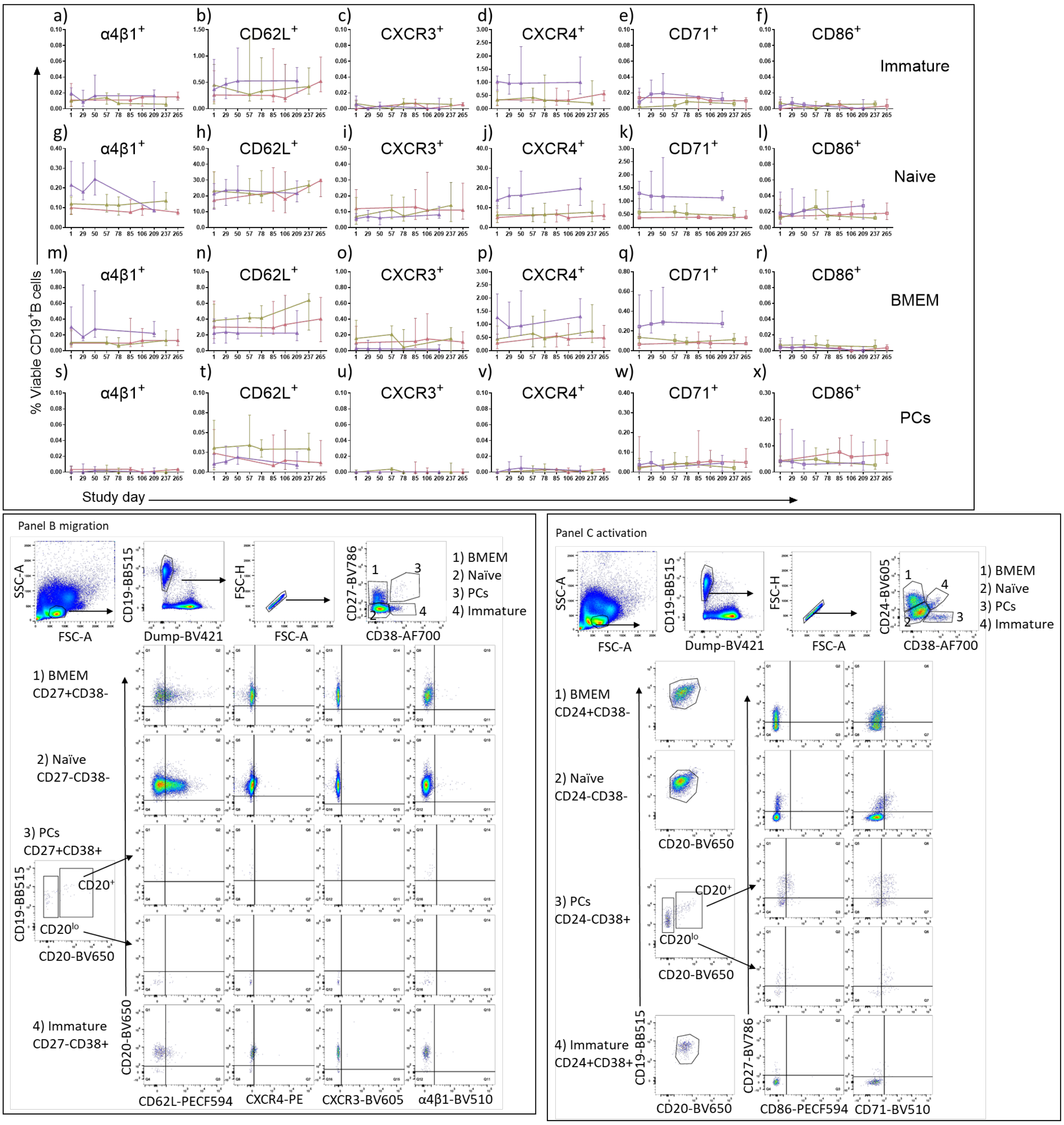

Supplementary figure 23: Core B cell migration and activation marker kinetics. The frequencies are expressed as percentage of total viable CD19+ B cells (a-x) with Group 1 (purple), Group 2 (olive) and Group 3 (coral). Data are expressed as median +/-IQ. B cell subset expression of a4β1, CD62L, CXCR3, CXCR4, CD71 and CD86 are show for Immature(a-f), Naïve(g-l), BMEM (m-r) and PCs (s-x). An example of the gating strategies are shown for the migration markers (antibody Panel B) and activation markers (antibody panel C), using the antibody cocktails given in Supplementary Table 12, Supplementary Methods. Analysis was undertake using Flow Jo version 10.1.1.

Supplementary tables

Supplementary table 1: EBL2001, Cohort 1, Ex vivo B cell ELISpot frequencies EBOV-GP specific IgG-ASC. Data are expressed as ZEBOV-IgG-ASC/million PBMCs and are shown as, number per group, per time poin (n), with Median, minimum to maximum and 25%-75% interquartile range. P-values two-sided Friedman Test, Dunn's multiple

*comparisons for changes post dose 1 (D1) to D9, D11, D13 or D15 and post dose 2 (D0) to D3, D5, D7 or D9. p-values *<0.05, **<0.01, ***,0.001, ****<0.0001.*

EBL2001 Cohort 1 ex vivo EBOV-GP specific IgG-ASC												
		Vaccine	Ad26.ZEBOV (dose 1)				MVA Dose 2	D0	D3	D5	D7	D9
Study day	All	D1	D9	D11	D13	D15	G1	D29	D32	D34	D36	D38
							G2	D57	D60	D62	D64	D66
							G3	D85	D88	D90	D92	D94
Group 1 MVA-BN-Filo D29	n=	8	8	7	8	8		8	8	7	7	6
	**Median**	**0.10**	**3.13**	**7.50**	**5.63 * p=0.0268**	**1.25**		**1.25**	**0.10**	**3.75**	**15.00**	**4.38**
	Min-max	(0.1-1.25)	(0.10-6.25)	(0.10-17.50)	(1.25-35.00)	(0.10-22.50)		(0.10-3.75)	(0.10-3.75)	(0.10-66.25)	(0.10-105.00)	(0.10-117.50)
	25%-75%	(0.1-1.25)	(0.70-5.63)	(0.10-12.50)	(1.89-17.50)	(0.10-10.63)		(0.10-3.44)	(0.10-3.13)	(0.10-10.00)	(3.75-78.75)	(0.10-62.19)
Group 2 MVA-BN-Filo D57	n=	9	9	9	9	9		9	9	9	9	9
	**Median**	**0.10**	**1.25**	**8.75 * p=0.0214**	**2.50**	**1.25**		**0.10**	**0.10**	**7.50**	**47.50 *** p=0.0001**	**18.75 ** p=0.0032**
	Min-max	(0.10-1.25)	(0.10-23.75)	(0.10-50.00)	(0.18-21.25)	(0.18-26.25)		(0.10-10.00)	(0.10-16.25)	(0.10-60.00)	(18.75-123.80)	(2.50-107.50)
	25%-75%	(0.10-0.10)	(0.10-15.63)	(1.88-28.75)	(0.68-10.63)	(0.68-3.13)		(0.10-2.50)	(0.10-3.75)	(0.10-51.25)	(29.38-81.88)	(7.50-76.88)
Group 3 MVA-BN-Filo D85	n=	9	9	9	9	9		9	8	8	8	8
	**Median**	**0.10**	**5.00**	**3.75**	**5.00**	**3.75**		**0.10**	**0.10**	**11.88 * p=0.0103**	**65.63 **** p=<0.0001**	**28.13 **** p=<0.0001**
	Min-max	(0.10-	(0.10-36.25)	(0.10-	(0.10-13.75)	(0.10-		(0.10-3.75)	(0.10-1.25)	(0.10-38.75)	(7.50-91.25)	(17.50-

		1.25)		11.25)		10.00)						106.30)
	25%-75%	(0.10-0.10)	(0.68-11.25)	(1.88-9.38)	(0.10-10.00)	(0.10-5.63)		(0.10-1.30)	(0.10-0.10)	(7.50-30.94)	(31.25-87.19)	(23.75-57.81)

*Supplementary table 2: EBL2001 Cohort 2 Ex vivo B cell ELISpot frequencies EBOV-GP specific IgG-ASC. Data are expressed as ZEBOV-IgG-ASC/million PBMCs and are shown as, number per group, per time point (n), with Median, minimum to maximum and 25%-75% interquartile range. P-values two-sided Kruskal-Wallis test, Dunn's Multiple comparsions: Values included in the table are for change from baseline (D1). Change from pre-dose 2 to d7 post dose 2 are: G1 D29 to D36 p=<0.0001; G2 D57 to D64 p=<0.0001; G3 D85 to D92 p=<0.0001. p-values *<0.05, **<0.01, ***,0.001, ****<0.0001.*

EBL2001 Cohort 2 ex vivo EBOV-GP						
	Vaccine	Ad26.ZEBOV Dose 1		MVA dose 2	D0	D7
Study day	All	D1	D11	G1	D29	D36
				G2	D57	D64
				G3	D85	D92
Group 1 MVA-BN-Filo D29	n=	28	24		32	31
	**Median**	**0.10**	**1.88** p=0.0027**		**0.10**	**32.50**** p=<0.0001**
	Min-max	(0.10-1.25)	(0.00-67.50)		(0.10-8.75)	(0.10-110.00)
	25%-75%	(0.10-0.10)	(0.39-8.13)		(0.10-3.44)	(2.50-47.50)
Group 2 MVA-BN-Filo D57	n=	24	22		28	30
	**Median**	**0.10**	**3.75** p=0.0061**		**0.10**	**54.38**** p=<0.0001**
	Min-max	(0.10-5.00)	(0.10-25.00)		(0.10-8.75)	(0.10-156.30)
	25%-75%	(0.10-0.10)	(0.10-11.25)		(0.10-2.50)	(20.94-82.50)
Group 3 MVA-BN-Filo D85	n=	24	23		30	27
	**Median**	**0.10**	**2.50**p=0.0031**		**0.10**	**62.50**** p=<0.0001**
	Min-max	(0.10-2.50)	(0.10-33.75)		(0.10-11.25)	(0.10-185.00)
	25%-75%	(0.10-0.10)	(0.10-6.25)		(0.10-1.25)	(21.25-98.75)

Supplementary table 3: EBL2001 Cohort 2 Memory B cell ELISpot frequencies of EBOV-GP specific IgG-BMEM. Data are expressed as EBOV-GP-BMEM/million cultured PBMCs. They are shown as, number per group, per time point (n=), with Median, minimum to maximum and 25%-75% interquartile range (p-values two-sided Friedman Test, Dunns Multiple comparison. Change from baseline, (D1) is shown in the table. Changes post dose 2 are: (G1 D29 to D209 p=0.0234). There was no significant change from D57(G2) or D85 (G3) . p-values *<0.05, **<0.01, ***,0.001, ****<0.0001.*

EBL2001 Cohort 2 EBOV-GP specific Memory B cell frequency						
	Vaccine	Ad26.ZEBOV Dose 1	MVA Dose 2	D0	D21	D180
Study day	All	D1	G1	D29	D50	D209
			G2	D57	D78	D237
			G3	D85	D109	D265
Group 1 MVA-BN-Filo D29	n=	13		13	13	13
	**Median**	**0.10**		**3.75**	**27.50** p=0.0029**	**56.25**** p=<0.0001**
	Min-max	(0.10-0.10)		(0.10-42.50)	(0.10-115.00)	(0.10-251.30)
	25%-75%	(0.10-0.10)		(0.63-14.38)	(13.75-39.38)	(11.88-149.40)
Group 2 MVA-BN-Filo D57	n=	13		13	13	13
	**Median**	**0.10**		**7.50**	**26.25** p=0.0065**	**42.50*** p=0.0005**
	Min-max	(0.10-0.10)		(0.10-153.80)	(0.10-211.30)	(0.10-268.80)
	25%-75%	(0.10-0.10)		(4.38-64.38)	(0.63-127.50)	(3.75-153.10)
Group 3 MVA-BN-Filo D85	n=	13		13	13	13
	**Median**	**0.10**		**35.00* p=0.0111**	**88.75**** p=<0.0001**	**25.00* p=0.0111**
	Min-max	(0.10-0.10)		(0.10-163.80)	(1.25-210.00)	(0.10-225.00)
	25%-75%	(0.10-0.10)		(2.50-87.50)	(15.63-107.50)	(5.63-100.60)

Supplementary table 4: PRISM Study: Memory B cell ELISpot frequencies of EBOV-GP specific IgG-BMEM. Data are expressed as EBOV-GP-BMEM/million cultured PBMCs. They are shown as, number per group, per time point (n=),

*with Median, minimum to maximum and 25%-75% interquartile range. (p-values two-sided Friedman Test, Dunn's Multiple comparison, change from baseline, D1). p-values *<0.05, **<0.01, ***,0.001, ****<0.0001.*

PRISM EBOV-GP specific Memory B cell frequency								
	Vaccine	Ad26.ZEBOV Dose 1	MVA dose 2	D0	D21	D180	V1	V2
Study day	All	D1	G1	D29	D50	D209	4 years post dose 2	6 months post V1
			G2	D57	D78	D237		
			G3	D85	D106	D265		
Group 1 MVA-BN-Filo D29	n=	6		6	6	6	6	6
	**Median**	**0.10**		**3.13**	**30.00**	**42.50* p=0.0234**	**40.63* p=0.0234**	**18.13**
	Min-max	(0.10-0.10)		(0.10-42.50)	(0.10-115.00)	(0.10-171.30)	(7.50-71.25)	(10.00-101.30)
	25%-75%	(0.10-0.10)		(1.88-19.06)	(18.75-67.19)	(15.00-97.19)	(14.06-64.69)	(10.00-61.88)
Group 2 MVA-BN-Filo D57	n=	6		6	6	6	6	6
	**Median**	**0.10**		**48.13**	**81.25**	**149.40** p=0.0043**	**48.13**	**46.88**
	Min-max	(0.10-0.10)		(3.75-153.80)	(0.10-157.50)	(3.75-268.80)	(15.00-206.30)	(16.25-248.80)
	25%-75%	(0.10-0.10)		(6.56-137.80)	(36.56-146.30)	(32.81-190.00)	(18.75-126.60)	(30.31-113.80)
Group 3 MVA-BN-Filo D85	n=	6		6	6	6	6	6
	**Median**	**0.10**		**25.00**	**55.00* p=0.0179**	**9.38**	**32.50*p=0.0136**	**32.50*p=0.0393**
	Min-max	(0.10-0.10)		(2.50-163.80)	(2.50-210)	(0.10-225.00)	(1.25-235.00)	(10.00-233.80)
	25%-75%	(0.10-0.10)		(2.50-95.31)	(12.81-125.6)	(1.88-95.63)	(20.00-138.40)	(11.88-107.20)

Supplementary table 5: EBL2002 Memory B cell ELISpot frequencies of EBOV-GP specific IgG-BMEM. Data are expressed as EBOV-GP-BMEM/million cultured PBMCs. They are shown as, number per group, per time point (n=), with Median, minimum to maximum and 25%-75% interquartile range. Participant samples were collected at study sites in Burkina Faso, Uganda and Kenya. Only samples from study groups 1 (MVA given at d29) and group 2 (MVA

*given at d57. P-values two-sided Kruskal-Wallis, Dunn's multiple comparison, change from baseline (D1) is shown in the table. Change post dose 2 for G2 D57 to D78 p=0.0014**. p-values *<0.05, **<0.01, ***,0.001, ****<0.0001.*

**EBL2002 EBOV-GP specific Memory B cell frequency**							
	Vaccine	Ad26.ZEBOV dose 1	MVA Dose 2	D0	D21	D180	D365
Study day	All	D1	G1	D29	D50	D180	D365
			G2	D57	D78	D180	D365
Group 1 MVA-BN-Filo D29	n=	8		8	8	7	8
	**Median**	**0.10**		**0.10**	**10.00**	**36.25**	**10.63**
	Min-max	(0.10-6.25)		(0.10-21.25)	(0.10-195.0)	(0.10-92.50)	(0.10-41.25)
	25%-75%	(0.10-1.25)		(0.10-0.96)	(0.70-125.30)	(0.10-75.00)	(0.10-29.06)
Group 2 MVA-BN-Filo D57	n=	11		11	11	11	10
	**Median**	**0.10**		**0.10**	**37.50 *** p=0.0005**	**28.75* p=0.0014**	**7.50**
	Min-max	(0.10-3.75)		(0.10-10.00)	(0.10-105.00)	(0.10-75.00)	(0.10-63.75)
	25%-75%	(0.10-2.50)		(0.10-3.75)	(18.75-87.50)	(3.75-50.00)	(0.10-32.50)

Supplementary methods

Antibodies used

BD Biosciences: mouse-anti-human CD19-BB515(HIB19), IgD-PE(IA6-2), CD10-PECF594 (HI10a), CD23-PECy7(M-L233), CD21-APC(B-Ly4), CD38-AF700(HIT2), CD43-APCH7(1G10), IgM-BV510 (G20-127), CD24-BV605(ML5), CD20-BV650(2H7), CD27-BV786(L128), CXCR4-PE(CD184, 12G5), CD62L-PECF594(L-Selectin, DREG-56), CXCR3-BV605 (CD183,IC6), a4B1-BV510(CD29, MAR4), CD11b-PECF594 (ICRF44), CD5-APCCy7(UCHT2), CD1d-BV510(CD1d42), CD86-PECF594(B70/7.2, 2331/FUN-1), CD40-BV510(5C3), CD69-BV605(FN50), PD1-BV786(CD279, EH12.1), CD3-BV421(SK7,Leu4), CD4-BV421(RPA-T4), CD14-BV421(MØP9), CD16-BV421(3G8), BD Horizon Fixable Viability Stain BV450, CD71-BV510(M-A712). Ebiosciences: mouse-anti-human CD138-APC (Syndcan-1, DL-101), CD3-eFluor450(OKT3), CD4-eFluor450(SK3), CD14- eFluor450(61D3), CD16- eFluor450(CB16), Fixable Viability Dye eFluor450.

Buffers used — Wash buffer: Automacs™ Running Buffer (0.5%BSA, Miltenyi) plus 50mM 2-Mercaptoethanol (gibco). Complete Medium (CM): RPMI supplemented with 10% FBS (batch tested) , Penicillin-Streptomycin, L-Glutamine, NEAA, Sodium Pyruvate, 50mM 2-Mercaptoethanol, Brilliant Violet Staining Buffer (BVB, BD Biosciences), CS&T beads, Sphero Rainbow particles

PBMCs removed from Liquid Nitrogen on to dry ice and then defrosted rapidly in complete medium (CM) plus benzonase (2µl in 10ml CM), at 37°C, in batches to minimise cell death. The defrosted cells were washed twice at 1000xg for 10 minutes and then resuspended in CM to count using a Millipore MUSE cell counter with Cell count and viability kit (Cytek, UK). For the memory B cell ELISpot assay the defrosted PBMCs were rested overnight at a concentration of $4x10^6$ cells/ml of CM at 37C/95% humidity/5% CO2. The following day the cells were pelleted, re-counted and resuspended at $2x10^6$ cells/ml CM for polyclonal expansion.

For flow cytometric phenotyping in Cohort 1, $4x10^6$ defrosted PBMCs were resuspended in 1ml Rinse buffer and 1µl of 1:2 dilution of BD Fixable Viability dye BV450 added per sample. The cells were stained for 10 mins, in dark at room temperature and then washed at 300xg for 10 minutes and then resuspended 400µl running buffer and 100ul ($1x10^6$ cells) added in quadruplate V-bottom plate wells, for staining.

For the Cohort 2 Phenotyping $3x10^6$ PBMCs were stained for viability as above and then washed and resuspended in 300µl running buffer and 100µl ($1x10^6$ cells) added in triplicate V-bottom wells for staining.

Antibody cocktails were prepared for 4 panels of antibodies (Cohort 1) and 3 panels of antibodies (Cohort 2), see tables i and ii, to allow core B cell subsets along with migration and activation status and expression of innate B cell associated markers to be assessed within each . A similar procedure was followed for the Cohort 1 TFH staining.

Cohort 1 Samples were run on a BD FACS Aria III, Blue-Red-Violet configuration and Cohort 2 samples were run on a BD Fortessa X20 Yellow/Green-Blue-Red-Violet configuration. All data were acquired using BD FACSDivaTM V8 software and analysed using Flow Jo v10.1 or Cytognos Infinicyt™ V1.8.

Supplementary table 6. Cohort 1 B cell Phenotyping antibody panels (see antibodies used for clones)

Aria Config	Filter	Panel A Core	Panel B migration	Panel C Innate	Panel D Activation
Gating strategy		Suppl.fig 16	Suppl.fig 17	Suppl.fig 18	Suppl.fig 19
B530/30	502LP	CD19-BB515	CD19-BB515	CD19-BB515	CD19-BB515
B585/42	556LP	IgD-PE	CXCR4-PE	IgD-PE	N/A
B616/23	610LP	CD10-PECF594	CD62L-PECF594	CD11b-PECF594	CD86-PECF594
B780/60	735LP	CD23-PECy7	N/A	CD23-PECy7	N/A
R660/20	N/A	CD21-APC	CD138-APC	CD21-APC	N/A

R730/45	690LP	CD38-AF700	CD38-AF700	CD38-AF700	N/A
R780/60	735LP	CD43-APCH7	N/A	CD5-APCCy7	N/A
V450/40	N/A	CD3-efluor450	CD3-efluor450	CD3-efluor450	CD3-efluor450
		CD4- efluor450	CD4- efluor450	CD4- efluor450	CD4- efluor450
		CD14- efluor450	CD14- efluor450	CD14- efluor450	CD14- efluor450
		CD16- efluor450	CD16- efluor450	CD16- efluor450	CD16- efluor450
		Ebio Vi- efluor450	Ebio Vi- efluor450	Ebio Vi- efluor450	Ebio Vi- efluor450
V510/50	502LP	IgM-BV510	α4β1-BV510	CD1d-BV510	CD40-BV510
V610/20	600LP	CD24-BV605	N/A	CD24-BV605	CD69-BV605
V670/30	640LP	CD20-BV650	CD20-BV650	CD20-BV650	CD20-BV650
V780/60	750LP	CD27-BV786	CD27-BV786	CD27-BV786	PD1-BV786

Supplementary Table 7. Cohort 2 B cell Phenotyping antibody panels (see antibodies uses for clones)

Fortessa Config	Filter	Panel A Core	Panel B migration	Panel C Activation
Gating strategy		Suppl.fig 20+21	Suppl.fig 22	Suppl. fig 23
B530/30	502LP	CD19-BB515	CD19-BB515	CD19-BB515
YG585/15	N/A	IgD-PE	CXCR4-PE	IgD-PE
YG610/20	610LP	CD10-PECF594	CD62L-PECF594	CD86-PECF594
YG780/60	735LP	CD23-PECy7	CD23-PECy7	CD23-PECy7
R670/30	N/A	CD21-APC	CD21-APC	CD21-APC
R730/45	690LP	CD38-AF700	CD38-AF700	CD38-AF700
R780/60	755LP	CD43-APCH7	CD5-APCCy7	CD5-APCCy7
V450/40	N/A	CD3-BV421	CD3-BV421	CD3-BV421
V450/40		CD4-BV421	CD4-BV421	CD4-BV421
V450/40		CD14-BV421	CD14-BV421	CD14-BV421
V450/40		CD16-BV421	CD16-BV421	CD16-BV421
V450/40		BD Vi-BV450	BD Vi-BV450	BD Vi-BV450
V525/50	502LP	IgM-BV510	α4β1-BV510	CD71-BV510

V610/20	600LP	CD24-BV605	CXCR3-BV605	CD24-BV605
V670/30	640LP	CD20-BV650	CD20-BV650	CD20-BV650
V780/60	750LP	CD27-BV786	CD27-BV786	CD27-BV786
