# Supplementary material for: Prediction and characterisation of the human B cell response to a heterologous two-dose Ebola vaccine
Source: Nat Commun. 2025 Jul 9;16:6331. doi: 10.1038/s41467-025-61571-x (PMC12241346; doi:10.1038/s41467-025-61571-x)
Supplement: Supplementary file 2 — Description of Additional Supplementary Files [file 41467_2025_61571_MOESM2_ESM.pdf]

# Description of Additional Supplementary Files

File Name: Supplementary Data 1.
Description: Table showing the two-sided Spearman correlations between the expression levels of differentially expressed genes (DEGs) post-first vaccine dose and EBOV glycoprotein-binding antibodies ZEBOV-specific IgG antibody levels measured after vaccination.

File Name: Supplementary Data 2.
Description: Table showing the two-sided Spearman correlations between the expression levels of differentially expressed genes (DEGs) post-second vaccine dose and EBOV glycoprotein-binding antibodies levels measured after vaccination.

File Name: Supplementary Data 3.
Description: Table of the cell type counts per sample in the single cell RNA-sequencing data

File Name: Supplementary Data 4.
Description: Table of significant enriched pathways (FDR <0.05) from gene set enrichment analysis using a one-sided permutation-based Kolmogorov–Smirnov statistic (C5 biological process) of differentially expressed genes in pseudobulk plasma cells at baseline compared with 10 days after the first dose of vaccine.

File Name: Supplementary Data 5.
Description: Table of significant enriched pathways (FDR <0.05) from gene set enrichment analysis using a one-sided permutation-based Kolmogorov–Smirnov statistic (C5 biological process) of differentially expressed genes in pseudobulk plasma cells at baseline compared with 7 days after the second dose of vaccine.
