# Supplementary material for: Prediction and characterisation of the human B cell response to a heterologous two-dose Ebola vaccine
Source: Nat Commun. 2025 Jul 9;16:6331. doi: 10.1038/s41467-025-61571-x (PMC12241346; doi:10.1038/s41467-025-61571-x)
Supplement: Supplementary file 8 — Reporting Summary [file 41467_2025_61571_MOESM8_ESM.pdf]

nature portfolio

Corresponding author(s): Daniel O'Connor

Last updated by author(s): Jun 23, 2025

Reporting Summary

Nature Portfolio wishes to improve the reproducibility of the work that we publish. This form provides structure for consistency and transparency in reporting. For further information on Nature Portfolio policies, see our Editorial Policies and the Editorial Policy Checklist.

Statistics

For all statistical analyses, confirm that the following items are present in the figure legend, table legend, main text, or Methods section.

n/a	Confirmed	
☐	☒	The exact sample size (n) for each experimental group/condition, given as a discrete number and unit of measurement
☐	☒	A statement on whether measurements were taken from distinct samples or whether the same sample was measured repeatedly
☐	☒	The statistical test(s) used AND whether they are one- or two-sided<br>*Only common tests should be described solely by name; describe more complex techniques in the Methods section.*
☐	☒	A description of all covariates tested
☐	☒	A description of any assumptions or corrections, such as tests of normality and adjustment for multiple comparisons
☐	☒	A full description of the statistical parameters including central tendency (e.g. means) or other basic estimates (e.g. regression coefficient) AND variation (e.g. standard deviation) or associated estimates of uncertainty (e.g. confidence intervals)
☐	☒	For null hypothesis testing, the test statistic (e.g. F, t, r) with confidence intervals, effect sizes, degrees of freedom and P value noted<br>*Give P values as exact values whenever suitable.*
☒	☐	For Bayesian analysis, information on the choice of priors and Markov chain Monte Carlo settings
☒	☐	For hierarchical and complex designs, identification of the appropriate level for tests and full reporting of outcomes
☒	☐	Estimates of effect sizes (e.g. Cohen's d, Pearson's r), indicating how they were calculated

Our web collection on statistics for biologists contains articles on many of the points above.

Software and code

Policy information about availability of computer code

Data collection	The R Project for Statistical Computing software
Data analysis	STAR (version 2.6.1d), HTSeq (version 0.11.1), RNA2HLA (version 1.1), R packages (ggpubr version 0.6.0, gridExtra version 2.3, caret version 6.9-94, plotly 4.10.4, factoextra vesiojn 1.0.7, ggplot2 version 3.5.1, edgeR version 4.2.1, limma version 3.60.4)

For manuscripts utilizing custom algorithms or software that are central to the research but not yet described in published literature, software must be made available to editors and reviewers. We strongly encourage code deposition in a community repository (e.g. GitHub). See the Nature Portfolio guidelines for submitting code & software for further information.

Data

Policy information about availability of data

All manuscripts must include a data availability statement. This statement should provide the following information, where applicable:

- Accession codes, unique identifiers, or web links for publicly available datasets
- A description of any restrictions on data availability
- For clinical datasets or third party data, please ensure that the statement adheres to our policy

The gene expression datasets described in this manuscript are available in the Gene Expression Omnibus (GSE; https://www.ncbi.nlm.nih.gov/geo/query/acc.cgi?acc=GSE273114 , reviewer token gburckikpvorhgd

Research involving human participants, their data, or biological material

Policy information about studies with human participants or human data. See also policy information about sex, gender (identity/presentation), and sexual orientation and race, ethnicity and racism.

Reporting on sex and gender	Biological sex information has been collected in the trial and reported previous — https://doi.org/10.1016/S1473-3099(20)30515-6. No sex or gender analysis was carried out, as not powered for this analysis but individual-level data are provided to contribute to future meta-analyses etc.
Reporting on race, ethnicity, or other socially relevant groupings	Race and ethnicity of the participants have been collected as a part of the trial, but was not reported in the current manuscript
Population characteristics	Participants were eligible if they were aged 18–65 years and healthy, as judged by investigators on the basis of a physical examination, an electrocardiogram, laboratory assessments, and a clinical history at screening. All women were required to have a negative pregnancy test before enrolment and before each vaccination, and were required to use approved effective methods of contraception during the study.
Recruitment	For EBL2001 Participants in this manuscript were recruited at Oxford, UK via mail out and screened if they were 18-65 years of age and judged to be healthy following physical exam, electrocardiogram and laboratory assessment as described in PMID:33217361.<br>EBL2002: Information was shared through community meetings, posters, and public conferences where volunteers were invited to study sites. For health adults, age was stratified as 18 to 50 years for samples used in this manuscript. Full recruitment details are described in PMID:34714820.
Ethics oversight	EBL2001: The study protocol was approved by the French national Ethics Committee (CPP Ile de France III; 3287), the French Medicine Agency (150646A-61), the UK Medicines and Healthcare Products Regulatory Agency (MHRA), and the UK National Research Ethics Service (South Central, Oxford; A 15/SC/0211). The study was done according to the current Declaration of Helsinki and the Good Clinical Practice guidelines. All participants provided written informed consent before enrollment. The study is registered at ClinicalTrials.gov, NCT02416453, and EudraCT, 2015-000596-27.<br><br>EBL2002: Samples used in this manuscript were obtained under the Phase II randomised, observer-blind, placebo-controlled study was conducted in 7 sites in Africa [Burkina Faso (Bobo-Dioulasso, Banfora); Coˆte d'Ivoire (Abidjan, Toupah/ Ousrou); Kenya (Nairobi); Uganda (Masaka, Kampala)] between November 2015 and Feb- ruary 2019. The protocol was approved by local and national independent Ethics Commit- tees and Institutional Review Boards, and the study was done according to the Declaration of Helsinki and International Conference on Harmonisation Good Clinical Practice Guide- lines. All adult participants supplied written informed consent before enrollment. An independent data monitoring committee was established to assess the safety data regularly during the study. The study was registered at ClinicalTrials.gov NCT02564523. Described in PMID:34714820.

Note that full information on the approval of the study protocol must also be provided in the manuscript.

Field-specific reporting

Please select the one below that is the best fit for your research. If you are not sure, read the appropriate sections before making your selection.

☒ Life sciences ☐ Behavioural & social sciences ☐ Ecological, evolutionary & environmental sciences

For a reference copy of the document with all sections, see nature.com/documents/nr-reporting-summary-flat.pdf

Life sciences study design

All studies must disclose on these points even when the disclosure is negative.

Sample size	Sample size the exploratory analyses included in this manuscript was determined by the samples available for analysis. Sample size calculations for the Clinical Trial primary endpoints are included in the the Clinical Trial manuscripts EBL2001 PMID:33217361 and EBL2002 PMID:34714820
Data exclusions	No data were excluded other than described in the manuscript.
Replication	N/A
Randomization	For the exploratory analysis included in this manuscript there was no further randomisation. For the main clinical trial and sample collection, randomisation occurred as part of the main clinical trial recruitment process and is described in the Clinical Trial manuscripts EBL2001 PMID:33217361 and EBL2002 PMID:34714820
Blinding	EBL2001: Was a Randomised, observer-blind, placebo-controlled, phase 2 trial. Cohort 1 was open label while for Cohort 2 study site personnel, sponsor personnel, and participants were masked to vaccine allocation until all participants in these cohorts had completed the post-MVA-BN-Filo vaccination visit at 6 months or had discontinued the trial as described in PMID:33217361.<br><br>EBL2002: Phase II randomised, observer-blind, placebo-controlled study. Participants, investigators, and study staff remained blinded to the allocation of investigational products throughout the study. Vaccines and placebo were prepared by a site pharmacist who was the only

unblinded member of staff. The pharmacist received the randomisation number and allocated the right study vaccine to the participant. Masking tape was used to cover the dispensing syringes containing the study vaccine/placebo allocated to each study participant, as described in PMID:34714820

Reporting for specific materials, systems and methods

We require information from authors about some types of materials, experimental systems and methods used in many studies. Here, indicate whether each material, system or method listed is relevant to your study. If you are not sure if a list item applies to your research, read the appropriate section before selecting a response.

Materials & experimental systems

n/a	Involved in the study
☐	☒ Antibodies
☒	☐ Eukaryotic cell lines
☒	☐ Palaeontology and archaeology
☒	☐ Animals and other organisms
☐	☒ Clinical data
☒	☐ Dual use research of concern
☒	☐ Plants

Methods

n/a	Involved in the study
☒	☐ ChIP-seq
☐	☒ Flow cytometry
☒	☐ MRI-based neuroimaging

Antibodies

Antibodies used

EVOLVE Cohort 1 Plasma cell analysis Panels -2016
Antibody Clone Product company Cohort 1 lot
CD19-BB515 HIB19 564456 BD Biosciences 5267592, 6014888,7075532
IgD-PE IA6-2 555779 BD Biosciences N/A
CXCR4-PE 12G5 555974 BD Biosciences 5260751
CD10-CFPE594 HI 10a 562396 BD Biosciences 5109932
CD62L-CFPE594 DREG-56 562301 BD Biosciences 5212645
CD11b-PECF594 ICRF44 (or 44) 562399 BD Biosciences 5030566
CD86-PECF594 2331 (FUN-1) 562390 BD Biosciences 4227758, 5196869
CD23-PECy7 M-L233 561167 BD Biosciences 5086929
CD21-APC B-Ly4 559867 BD Biosciences 5295821
CD138-APC DL-101 17-1389-42 eBiosciences E16209-104
CD38-AF700 HIT2 560676 BD Biosciences 4279747, 5289935
CD43-APCH7 1G10 655407 BD Biosciences 5306995
CD5-APCCy7 UCHT2 563516 BD Biosciences 5093932, 5288787
CD3-efluor450 OKT3 48-0037-42 eBiosciences E08482-1634
CD4-efluor450 SK3 (SK-3) 48-0047-42 eBiosciences E10494-1636, 4295630
CD14-efluor450 61D3 48-0149-42 eBiosciences E08492-1636, 4293193
CD16-efluor450 ebioCB16(CB16) 48-0168-42 eBiosciences 4278011
EbioVi efluor450 N/A 65-0863-18 eBiosciences N/A
IgM-BV510 G20-127 563113 BD Biosciences 4311997, 5267743, 6063603
a4B1(CD29)-BV510 MAR4. 563513 BD Biosciences 5068756, 5124599
CD1d-BV510 CD1d42(or 42.1) 563506 BD Biosciences 4164978, 5351575
CD40-BV510 5C3 563456 BD Biosciences 5134887, 7096533
CD24-BV605 ML5 562788 BD Biosciences 5163746, 5309804
CD69-BV605 FN50 562989 BD Biosciences 5244877
CD20-BV650 2H7 563780 BD Biosciences 5309636, 7079596
CD27-BV786 L128 563327 BD Biosciences 5212906, 5329693
PD1(CD279)-BV786 EH12.1 (EH12) 563789 BD Biosciences 5253586, 5149817, 6032898

EVOLVE Cohort 2 BMEM analysis Panels 2019
Antibody Clone Product company Cohort 2 lot
CD19-BB515 HIB19 564456 BD Biosciences 912583, 8248825
IgD-PE IA6-2 555779 BD Biosciences N/A
CXCR4-PE 12G5 555974 BD Biosciences 5260751
CD10-CFPE594 HI 10a 562396 BD Biosciences 5109932
CD62L-CFPE594 DREG-56 562301 BD Biosciences 5212645
CD86-PECF594 2331 (FUN-1) 562390 BD Biosciences 4227758, 5196869
CD23-PECy7 M-L233 561167 BD Biosciences 8284750, 7194655
CD21-APC B-Ly4 559867 BD Biosciences N/A
CD38-AF700 HIT2 560676 BD Biosciences 9100660
CD43-APCH7 1G10 655407 BD Biosciences N/A
CD5-APCCy7 UCHT2 563516 BD Biosciences 7250708

CD3-BV421 SK7(Leu 4) 563798 BD Biosciences 9098859
CD4-BV421 RPA-T4 562424 BD Biosciences N/A
CD14-BV421 MØP9 563743 BD Biosciences 9123846
CD16-BV421 3G8 562874 BD Biosciences 8183519
BD Fixable Viability Dye BV450 NA 562247 BD Biosciences 8194969
IgM-BV510 G20-127 563113 BD Biosciences 7089511, 9051699
a4B1(CD29)-BV510 MAR4. 563513 BD Biosciences 9170841
CD71-BV510 M-A712 743305 BD Biosciences 9151606
CD24-BV605 ML5 562788 BD Biosciences 9044725
CXCR3(CD183)-BV605 IC6 564032 BD Biosciences 7068545
CD20-BV650 2H7 563780 BD Biosciences 7342749
CD27-BV786 L128 563327 BD Biosciences 7303868

EVOLVE Cohort 1 TFh
Antibody Clone Product company Cohort 1 lot
CD25-BB515 M-A251 565096 BD Biosciences 6188758
ICOS(CD278)-PE C398.4A 313507 Biolegend B221869
PD1(CD279)-PE-610 eBioJ105 61-2799-41 eBIOSCIENCE/INVITROGEN 4324105
CD8a-PerCPCy5.5 HIT8a 300923 Biolegend B216128
CCR6(CD196)-PECy7 G034E3 353417 Biolegend B232625
CXCR3(CD183)-AF647 G025H7 353712 Biolegend B204394
CD4-AF700 OKT4 317426 Biolegend B224470
CCR7(CD197)-APCCy7 G043H7 353212 Biolegend B226224
CXCR5(CD185)-BV421 J252D4 356919 Biolegend B232067
Aqua-BV510 N/A 423101 Biolegend NA
CCR4(CD194)-BV605 L291H4 359417 Biolegend B208697
CD3-BV650 OKT3 317323 Biolegend B215923
CD45RA-BV711 HI100 304137 Biolegend B220066
CD38-BV786 HIT2 563964 BD Biosciences 7089805, 5309625

Validation

Each antibody was titrated to determine the optimal concentration on freshly isolated PBMCs and then the compensation matrix was tested and sent to BD Applications specialist for advice on final compensation values. The whole panel was then tested on PBMCs that had been stored in Liquid Nitrogen, defrosted and rested for four hours prior to staining. Antibodies for anti-IgM, anti-CD20 and anti-CD27 on B cell panels required re-optimisation on defrosted PBMCs, with an increase in concentration required to regain the staining profile from the fresh PBMCs. Once the panels were optimised, Mean Fluorescence Intensity for each channel was set against target values prior to each run, using Spherocyte rainbow 8-Peak beads. MFIs were set to target values for each run and recorded. The Compensation was carried out on freshly stained compensation beads prior to running samples.

Clinical data

Policy information about clinical studies
All manuscripts should comply with the ICMJEguidelines for publication of clinical research and a completedCONSORT checklist must be included with all submissions.

Clinical trial registration

NCT02416453

Study protocol

study protocol was registered at ClinicalTrials.gov,
NCT02416453, and EudraCT, 2015-000596-27.

Data collection

Enrolment was between June 23, 2015, and April 27, 2016,

Outcomes

The descriptive safety and tolerability analysis of the study vaccines was the primary outcome of the clinical trial

Plants

Seed stocks

N/A

Novel plant genotypes

N/A

Authentication

N/A

Flow Cytometry

Plots

Confirm that:

- [x] The axis labels state the marker and fluorochrome used (e.g. CD4-FITC).
- [x] The axis scales are clearly visible. Include numbers along axes only for bottom left plot of group (a 'group' is an analysis of identical markers).
- [ ] All plots are contour plots with outliers or pseudocolor plots.
- [x] A numerical value for number of cells or percentage (with statistics) is provided.

Methodology

Sample preparation	Peripheral Blood Mononuclear cells (PBMCs) were isolated and frozen within 6 hours of venepuncture as part of the clinical trial procedures. The samples were stored in Liquid Nitrogen until analysis [all time points for a participant were run within a single assay].
Instrument	For Cohort 1 plasma cell phenotyping and for B cells sorted for input into single cell squencing, data were obtained using a FACS Aria III (Blue_Red_Violet) instrument<br>For Cohort 2 B cell phenotyping, data were obtained using a Fortessa X20 (Blue_Red_Violet_Yellow/Green) instrument
Software	FACS Diva version 7.0, Flow Jo version 10.0 and InfinicytTM Version 1.8.
Cell population abundance	B cells sorted for single cell sequencing: Pre sort the %viable CD19+ cells ranged from 7.74% to 19.5% and following sorting the range was 60.2% to 79.9%. Total viable CD19+ cells collected ranged from 1.40x105 to 3.07x105 cells.<br>For B cell phenotyping
Gating strategy	The gating strategies for Plasma cell characterisation, Memory B cell Phenotying and TfH phenotyping are included in the manuscript and supplementary information.<br><br>PBMCs sorted for B cell single cell analysis:<br>PBMCs were defrosted, washed (all in complete medium (CM), RPMI+10%FBS+2-Mecaptoethanol) and resuspended in 1ml CM. Cell count and viability of total defrosted sample was obtained from the MUSE cell counter = total cell counts x106 and viability prior to sort processing (Fig a+b, black squares). PBMCs were then washed in PBS to remove proteins from buffer and then suspended in 1ml PBS+2-mercaptoethanol and stained with fixable viability dye-BV450 (1ul in 1ml,10 mins at room temp, in the dark). Cells were pelleted at 1800rpm/5 mins, Pellet resuspended in 100ul of cold, PBS-EDTA+0.5%BSA+2-Mercaptoethanol and stained with 30ul of CD19-BB515 for 30 minutes (on ice, in the dark). Cells resuspended in 5ml, cold PBS-EDTA+0.5%BSA+2-Mercaptoethanol and washed at 1800rpm for 5 mins. Final resuspension in 500ul cold PBS-EDTA+0.5% BSA+2-Mercaptoethanol. Defrosting and staining took about 2 hours for 6 samples. Samples were run on FACS ARIA sorter (following CST calibration and compensation). The entire sample was sorted at <10,000 events per second on stringent gates for lymphocytes (Fig d) into singlets (Fig c) into viable, CD19+ B cells (Fig e+f) (took 25-20 minutes per sample). 20ul of sorted cells were retained to compare the purity of unsorted (blue) vs sorted (green) fractions obtained for 10X. Pre sort the % viable CD19+ cells ranged from 7.74% to 19.5% and following sorting the range was 60.2% to 79.9%. The data for the B cell sorting is not currently included in the supplementary data. A JPEG of the figure is attached with this form.

- [x] Tick this box to confirm that a figure exemplifying the gating strategy is provided in the Supplementary Information.
